# Pyroptosis: the dawn of a new era in endometrial cancer treatment

**DOI:** 10.3389/fonc.2023.1277639

**Published:** 2023-10-30

**Authors:** Tian Peng, Chi Zhang, Wen-Jun Chen, Xue-Fei Zhao, Wei-Bo Wu, Wei-Ji Yang, Ruo-Jia Liang

**Affiliations:** ^1^ The First School of Clinical Medicine, Zhejiang Chinese Medical University, Hangzhou, China; ^2^ Sir Run Run Shaw Hospital, Zhejiang University, Hangzhou, China; ^3^ School of Nursing, Hangzhou Medical College, Hangzhou, Zhejiang, China; ^4^ Department of Gynaecology, The First Affiliated Hospital of Zhejiang Chinese Medical University (Zhejiang Provincial Hospital of Traditional Chinese Medicine), Hangzhou, Zhejiang, China

**Keywords:** endometrial cancer, treatment, pyroptosis, molecular mechanism, gasdermins

## Abstract

Endometrial cancer (EC) is a malignancy of the inner epithelial lining of the uterus. While early-stage EC is often curable through surgery, the management of advanced, recurrent and metastatic EC poses significant challenges and is associated with a poor prognosis. Pyroptosis, an emerging form of programmed cell death, is characterized by the cleavage of gasdermin proteins, inducing the formation of extensive gasdermin pores in the cell membrane and the leakage of interleukin-1β (IL-1β) and interleukin-18 (IL-18), consequently causing cell swelling, lysis and death. It has been found to be implicated in the occurrence and progression of almost all tumors. Recent studies have demonstrated that regulating tumor cells pyroptosis can exploit synergies function with traditional tumor treatments. This paper provides an overview of the research progress made in molecular mechanisms of pyroptosis. It then discusses the role of pyroptosis and its components in initiation and progression of endometrial cancer, emphasizing recent insights into the underlying mechanisms and highlighting unresolved questions. Furthermore, it explores the potential value of pyroptosis in the treatment of endometrial cancer, considering its current application in tumor radiotherapy, chemotherapy, targeted therapy and immunotherapy.

## 1 Introduction

Endometrial cancer (EC) is the most common female reproductive system malignancy in developed countries (1). From 1990 to 2019, Overall incidence has risen by 132% (2). In 2020, there were 417,000 new cases and 97,000 deaths of EC globally, predominantly observed in women aged 65 to 75. Socioeconomic, racial disparities and geographic differences largely determine incidence and mortality of EC (1, 3). The high prevalence of EC is associated with several risk factors, including population aging, exposure to endogenous or exogenous hormones, high body mass index (BMI), metabolic syndrome and epigenetic alterations (3, 4).

Abnormal uterine bleeding is the most common clinical symptom of EC (5), which ensures more than 60% of EC can be diagnosed at an early stage (6). For early-stage EC, the main treatment is surgery, and total hysterectomy with bilateral salpingo-oophorectomy (BSO) is standard of care. Depending on stage of disease and other risk factors, adjuvant radiotherapy and/or chemotherapy (carboplatin in combination with paclitaxel) can be used to reduce risk of recurrence (7). Over the past decade, four molecular subtypes have been identified through The Cancer Genome Atlas (TCGA)- polymerase ϵ ultramutated (POLEmut), mismatch repair-deficient (dMMR)/microsatellite instability-high (MSI-H), copy number low(CNL)/no specific molecular profile (NSMP) and copy number high (CNH)/p53abn-which significantly improved the prognosis of EC with hormone therapy, immunotherapy and targeted therapy (8, 9). While the 5-year overall survival (OS) exceeds 80% in early stage EC (10), it is only 17% and 15% in patients of stage IVA and IVB EC (11), which pose a serious threat to women’s health and well-being. Therefore, gaining a deeper understanding of the molecular changes occurring during the progression of EC and identifying potential biomarkers and therapeutic targets hold vital clinical significance. These endeavors can facilitate early screening and further enhance the prognosis of EC patients.

Pyroptosis is a recently discovered form of cell death that can be triggered by microbial infection and endogenous stimuli, which leads to the cleavage of gasdermins (GSDMs) and the formation of gasdermin pores on the cell membrane, resulting in cell lysis and death along with the leakage of intracellular content (12). Over the past 20 years, the interesting and important biological phenomenon of pyroptosis has attracted many scholars to investigate its corresponding molecular mechanisms. To date, not only in monocyte macrophages but also in neutrophils and dendritic cells (13), it has been discovered that pyroptosis can be induced by Salmonella (14), Shigella flexneri (15), Yersinia, Listeria (16), Vibrio cholerae and Pseudomonas aeruginosa (17). Pyroptosis plays a significant role in maintaining body homeostasis and certain diseases, including infectious diseases (18, 19), neurodegenerative diseases (20, 21), and metabolic diseases (22, 23). Moreover, regulating pyroptosis has been found to promote cancer cell death (24) and inhibit tumor progression in various types of cancers such as hepatocellular carcinoma (25), lung cancer (26), breast cancer (27), and ovarian cancer (28). Currently, there is growing evidence for an association between pyroptosis and EC. Therefore, this article aims to summarize the mechanisms underlying the anti-tumor effects of pyroptosis, with a specific focus on its role in the initiation and progression of EC. Additionally, it explores the potential application of pyroptosis in the treatment of EC.

## 2 Overview of pyroptosis

### 2.1 pyroptosis and cell death

Cell death is a crucial process for maintaining body homeostasis and suppressing the uncontrolled growth of tumor cells. It can be classified into regulated cell death (RCD) and accidental cell death (ACD) (29). The fully physiological form of RCD is referred to as programmed cell death (PCD) that plays a significant role in homeostasis maintenance (29). Resistance to apoptosis is recognized as a contributor to chemotherapy resistance in tumors, thus, exploring strategies to induce non-apoptotic programmed cell death holds promise for cancer therapy. Common types of RCD include apoptosis (29, 30), necroptosis (31), ferroptosis (32), autophagy (33) and pyroptosis. A brief summary of the distinguishing features of these different forms of RCD is presented in **Table 1**.

**Table 1:** Comparison of common types regulated cell death.

	Pyroptosis	Apoptosis	Necroptosis	Ferroptosis	Autophagy
Morphology	Swell	Shrink	Swell	Swell	Crescent-shaped
Membrane	Pore formation	Intact	Pore formation	Pore formation	Intact
Organelle	Intact/deformed	Intact	Swell	Smaller mitochondria	Engulfed by autophagosome
Remark(s)	Inflammasome	Apoptotic body	Necrosome	Autophagosome	Autophagosome
DNA	Random degradation	Ladder degradation	Random degradation	Random degradation	Random degradation
Caspase- dependent	√	√	×	×	×
IL-18, IL-1β release	√	×	√	√	×
Pore-forming cause	Gasdermin protein	No	MLKL	Iron-dependent	No
Inflammation	√	×	√	√	×

MLKL, mixed lineage kinase domain-like.

### 2.2 Characteristics of Pyroptosis

(1) Molecular Characteristics: executive protein (gasdermin family members, N-terminal) move to and oligomerize on the cell membrane, forming membrane pores that facilitate the secretion of mature interleukin (IL)-1β and IL-18. GSDMs exhibit a common autoinhibitory conformation in the inactive state, wherein gasdermins-C-terminal (GSDMs-CT, autoinhibition domain) binds to gasdermins-N-terminal (GSDMs-NT, pore-forming structure domain) (34). The GSDMs-NT which translocates on the cell membrane and forms extensive gasdermin pores on liposomes containing phosphoinositides, cardiolipin or natural polar lipid mixtures exhibit membrance-disrupting cytotoxicity (35, 36). Negatively charged conduits within the GSDM pores enable the discrimination between mature IL-1β and IL-18 and their precursors (37). Consequently, intracellular content including mature IL-1β, IL-18, ATP and high mobility group protein box 1(HMGB1) are released extracellularly through GSDMs pores (38). These substances play a pivotal role in recruiting various cells, such as macrophages and neutrophils, and contribute significantly to promoting inflammatory antibacterial immune responses (39, 40). (2) Morphological Characteristics: Gasdermin forms non-selective pores that allow the passage of different ions and water influx, resulting in bubble-like protrusions on cell membrane, DNA fragmentation, chromatin condensation as well as cell swelling, lysis and death.

In the context of cancer, pyroptosis appears to be a double-edged sword, simultaneously inducing robust inflammation and immune responses during cell death processes. Inflammation has been implicated in promoting tumorigenesis across various cancer types, including endometrial cancer. Pyroptosis can either promote or inhibit tumorigenesis by shaping the tumor microenvironment (TME) or facilitating cancer cell death (41). Therefore, gaining a comprehensive understanding of the specific mechanisms underlying pyroptosis is crucial before exploring its role in endometrial cancer. Traditionally, the pyroptotic pathway can be categorized into the canonical pathway (involving the typical inflammasome and caspase-1), the noncanonical pathway (involving caspase-4/5/11), and other pathways. The detailed description of the pyroptotic pathway is presented below:

## 3 Canonical pyroptotic pathway

**Figure 1:**
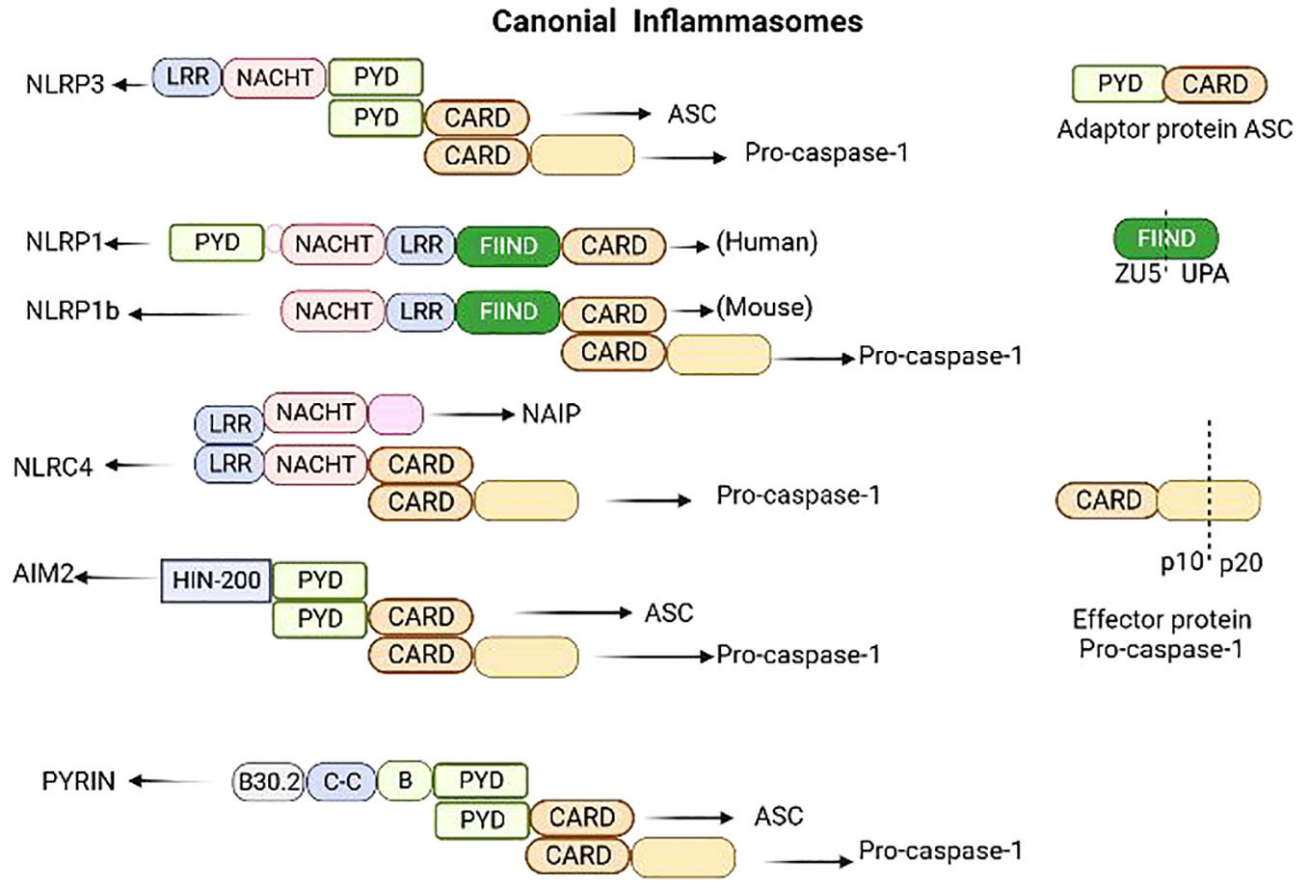
Structure of inflammasomes involved in canonical pyroptotic pathway. The inflammasomes primarily consists of cytoplasmic pattern recognition receptors (PRRs), also known as inflammasome sensors, apoptosis-associated speck-like protein (ASC)as the adaptor protein and pro-caspase-1 as the effector protein. Inflammasome sensors are cytosolic proteins which contain a pyrin domain (PYD) and/or a caspase-activation and recruitment domain (CARD). They may also contain a NACHT domain (synthesized by the abbreviations of four kinds of NLR members: NAIP, CIITA, HETE, TP1), a leucine-rich-repeat domain (LRR), a HIN-200 domain, a B30.2 domain, a coiled-coil domain (CC), a B-box domain (B) or a function-to-find domain (FIIND). ASC is primarily composed of an N-terminal pyrin domain (PYD) and a caspase activation and recruitment domain (CARD). The FIIND domain contains a ZU5 subdomain and an UPA subdomain.

The term “inflammasome” was introduced by Martinon et al. to describe a cytoplasmic multiprotein signal transducer found in activated immune cells that facilitates the activation of inflammatory caspases (42). The inflammasome primarily consists of cytoplasmic pattern recognition receptors (PRRs), apoptosis-associated speck-like protein (ASC) as the adaptor protein and pro-caspase-1 as the effector protein (43). PRRs, also known as inflammasome sensors, include members of the nucleotide-binding oligomerization domain (NOD) with leucine-rich repeat (LRR) protein family (NLR, NLRP1, NLRP3, and NLRC4), non-NLR receptors such as absent in melanoma 2 (AIM2) and pyrin (39) (**Figure 1**). NOD, also known as the NACHT domain, which is shared by the NLR family and play an important role in nucleic acid binding. ASC, a critical component, is primarily composed of an N-terminal pyrin domain (PYD) and a caspase activation and recruitment domain (CARD) (44).

**Figure 2:**
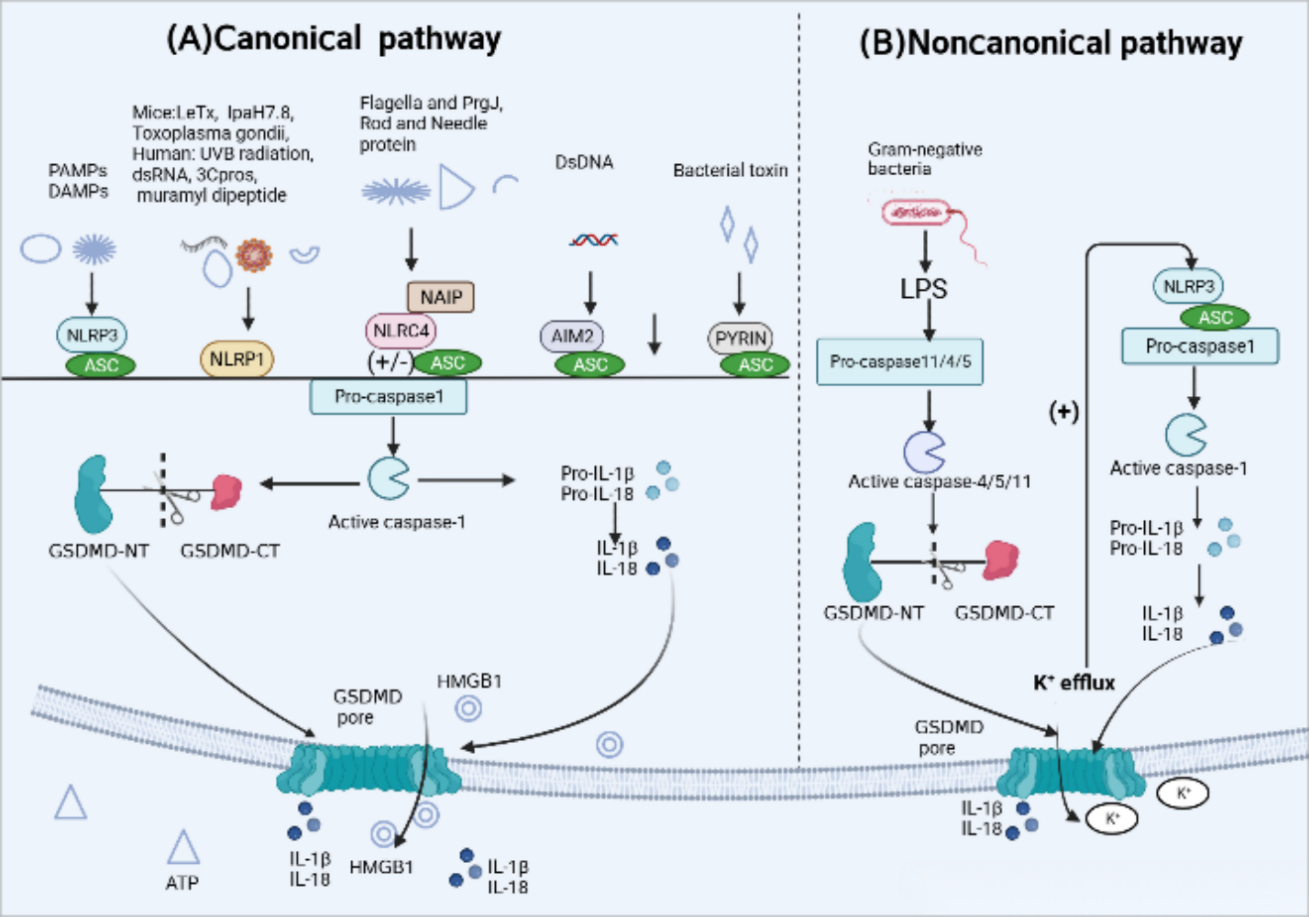
Activation mechanism of canonical and Noncanonical pyroptotic pathway. **(A)** Canonical inflammasome activation occurs in response to pathogen-associated molecular patterns (PAMPs) and host-derived danger-associated molecular pattern molecules (DAMPs). Responding to a variety of physiological or pathological changes, Inflammasome sensors, such as NLRP3, AIM2 and Pyrin, which contain a PYD may recruit adaptor protein apoptosis-associated speck-like protein (ASC) containing a caspase activation and recruitment domain (CARD) to mediate CARD–CARD interactions with effector protein pro-caspase-1. However, murine NLRP1b and NLRC4 contain a CARD and can interact directly with pro-caspase-1 without the ASC. After the above signal cascade response, inflammasomes activate pro-caspase-1 into active form. Activated caspase-1 cleaves pro-interleukin(IL)-1β and pro-IL-18 into their active forms. Caspase-1 also cleaves gasdermin D (GSDMD) to release the N-terminal domain (GSDMD-NT) which oligomerize on the cell membrane, forming membrane pores that induce the leakage of intracellular content inculding IL-1, IL-18, ATP and high mobility group protein box 1 (HMGB1) release and pyroptotic cell death. **(B)** Noncanonical pyroptotic pathway is mediated by human caspase-4/5 or murine caspase-11, which are activated by lipopolysaccharide (LPS) released from Gram-negative bacteria. Activated caspases cleave GSDMD into GSDMD-NT, leading to the formation of GSDMD pores. In addition, potassium(K+) efflux through those pores is enough to activate NLRP3 inflammasome, subsequently triggering caspase-1-dependent release of IL-1β and IL-18.

In response to infection or specific immune diseases, inflammasome sensors activated by various upstream signals, including pathogen-associated molecular patterns (PAMPs) as well as host-derived danger-associated molecular patterns (DAMPs) (13), initiate the assembly of inflammasomes. PRRs like NLRP3, AIM2 and PYRIN contain a PYD may recruit ASC to mediate CARD–CARD interactions with pro-caspase-1. However, murine NLRP1b and NLRC4 contain a CARD and can interact directly with pro-caspase-1 without the need of ASC (39, 44). Active caspase-1, also known as interleukin-converting enzyme, cleaves pro-IL-1β and pro-IL-18 to promote the production of mature IL-1β and IL-18. Meanwhile, it specifically cleaves the GSDMD domain, resulting in the GSDMD-NT fragments oligomerize on the cell membrane, forming membrane pores that induce cytokine release and pyroptotic cell death (44, 45) (**Figure 2**). The canonical pathways involve five major types of inflammasomes: NLRP3 inflammasome, NLRP1 inflammasome, NLRC4 inflammasome, AIM2 inflammasome, and PYRIN inflammasome.

### 3.1 Activation mechanism of the NLRP3 Inflammasome

**Figure 3:**
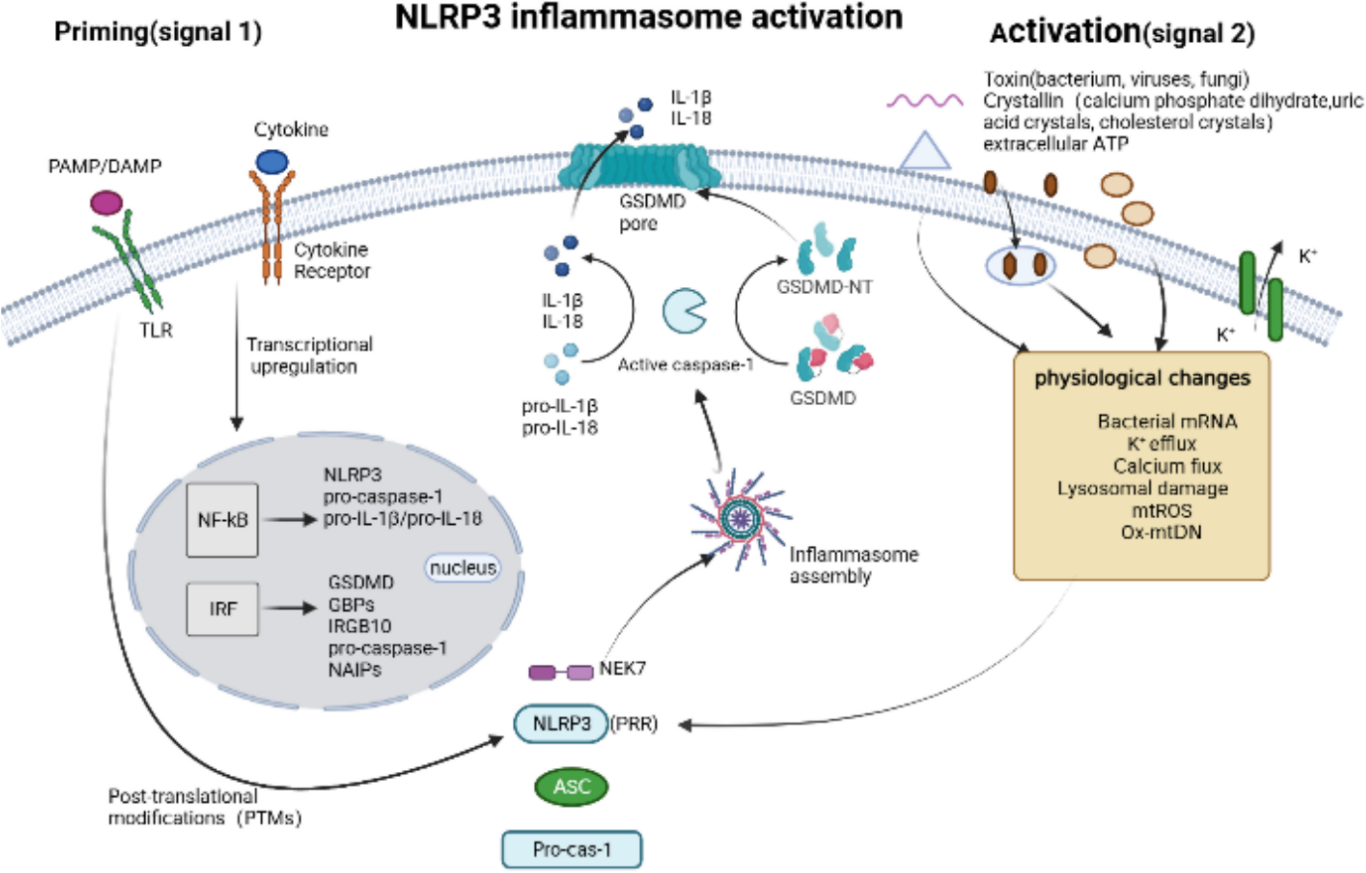
NLRP3 inflammasome activation. Canonical NLRP3 inflammasome activation begins with a priming signal, driven by Toll-like receptors (TLRs) or cytokine receptors, which facilitate the upregulation of components associated with pyroptosis, inculding NLRP3, pro-caspase-1, pro-IL-1β/18, GSDMD, GBPs and IRGB10, and regulate the post-translational modifications (PTMs). An activation signal is then initiated by pathogen-associated molecular patterns (PAMPs), such as toxins from bacterium, viruses and fungi, or danger-associated molecular pattern molecules (DAMPs), such as calcium phosphate dihydrate, uric acid crystals, cholesterol crystals and extracellular ATP, which induce physiological changes that are detected by NLRP3 and subsequently trigger the the assembly of NLRP3 Inflammasome via Human and murine NIMA-related kinase 7 (NEK7).

The NLRP3 inflammasome sensor, which is extensively studied as an intracellular sensor, consists of three domains: a LRR domain at the C-terminus, a central nucleotide-binding and oligomerization domain with ATPase activity called NACHT, and a PYD at the N-terminus (44). The basal expression of NLRP3 is insufficient to activate the inflammasome, and its activation involves two major steps (**Figure 3**): initiation and activation. Firstly, initiation occurs through signaling from Toll-like receptors (TLRs) or cytokine receptors, which promotes the transcription and translation of NLRP3 and cytokines (46). Subsequently, the NLRP3 receptor proteins assemble and oligomerize upon exposure to inflammasome activating factors such as PAMPs or DAMPs, which recruits ASC and pro-caspase-1, resulting in the self-regulation and activation of caspase-1 (39). These PAMPs consist of components derived from Gram-positive bacteria (16, 47), Gram-negative bacteria (15), viruses (18, 48), fungi (49–51), and microbial activators of protozoan pathogens. Additionally, DAMPs involve extracellular ATP (16), calcium phosphate dihydrate (52), uric acid crystals and cholesterol crystals (53). NLRP3 sensors were activated by PAMPs and DAMPs through various processes, including K^+^ efflux (54, 55), Ca^2+^ flux (56), Lysosomal damage (57), generation of mitochondrial reactive oxygen species (mtROS) (58, 59), release of oxidized mitochondrial DNA (Ox-mtDNA) (60).

In recent years, research on NLRP3 has focused on understanding its activation mechanism and the state in which it is activated. NLRP3 is primarily present in an autoinhibited state in cells, mainly in the cytoplasm and trans-Golgi apparatus (TGN), forming monomeric or oligomeric cage structures (61, 62). Chen et al. reported for the first time that various NLRP3-activating stimuli, regardless of their diverse sources, chemical compositions, and structural properties, can trigger a common cellular signal. Specifically, phosphatidylinositol 4-phosphate (PI4P) induces the transport and accumulation of NLRP3 to the specific disassembled trans-Golgi network structure (dTGN) (62). Zhang et al. further revealed that disruption of the endoplasmic reticulum-endosome membrane contact sites (EECS) leads to the accumulation of phosphatidylinositol 4-phosphate (PI4P) in lysosomes, which is crucial for NLRP3 inflammasome activation (63). However, the exact mechanism by which activators induce dTGN structure formation remains unclear. NEK7, a serine/threonine kinase localized in the microtubule organizing center, has been implicated in the assembly and activation of the NLRP3 inflammasome during mitotic interphase (64). Recently, Xiao et al. successfully reconstituted and stabilized the human NEK7-NLRP3-ASC inflammasome activated by nigericin both *in vivo* and *in vitro*. They analyzed the complex’s high-resolution electron microscope structure and discovered that NEK7 interacts with and competes with LRR domain of NLRP3. It is speculated that NEK7 may play a role in opening the self-inhibitory cage structure of NLRP3 (65).

### 3.2 Activation mechanism of NLRP1 Inflammasome

NLRP1 is among the earliest discovered pattern recognition receptors. Human NLRP1 comprises five domains: a PYD, a NACHT domain, a LRR domain, the function-to-find domain (FIIND), and a CARD. The regulatory mechanism of human NLRP1 remains unclear. Recent studies have demonstrated that human NLRP1 is capable of sensing double-stranded viral RNA (66), Ultraviolet B (UVB) radiation and 3C protease (3Cpros) derived from enteroviruses like human rhinovirus (HRV) (67). However, none of these PAMPs can activate the rodent NLRP1 inflammasome. Interestingly, only inhibitors of dipeptidyl peptidase 8 (DPP8) and DPP9 have been identified as capable of activating both rodent and human endogenous NLRP1 inflammasomes (68).

**Figure 4:**
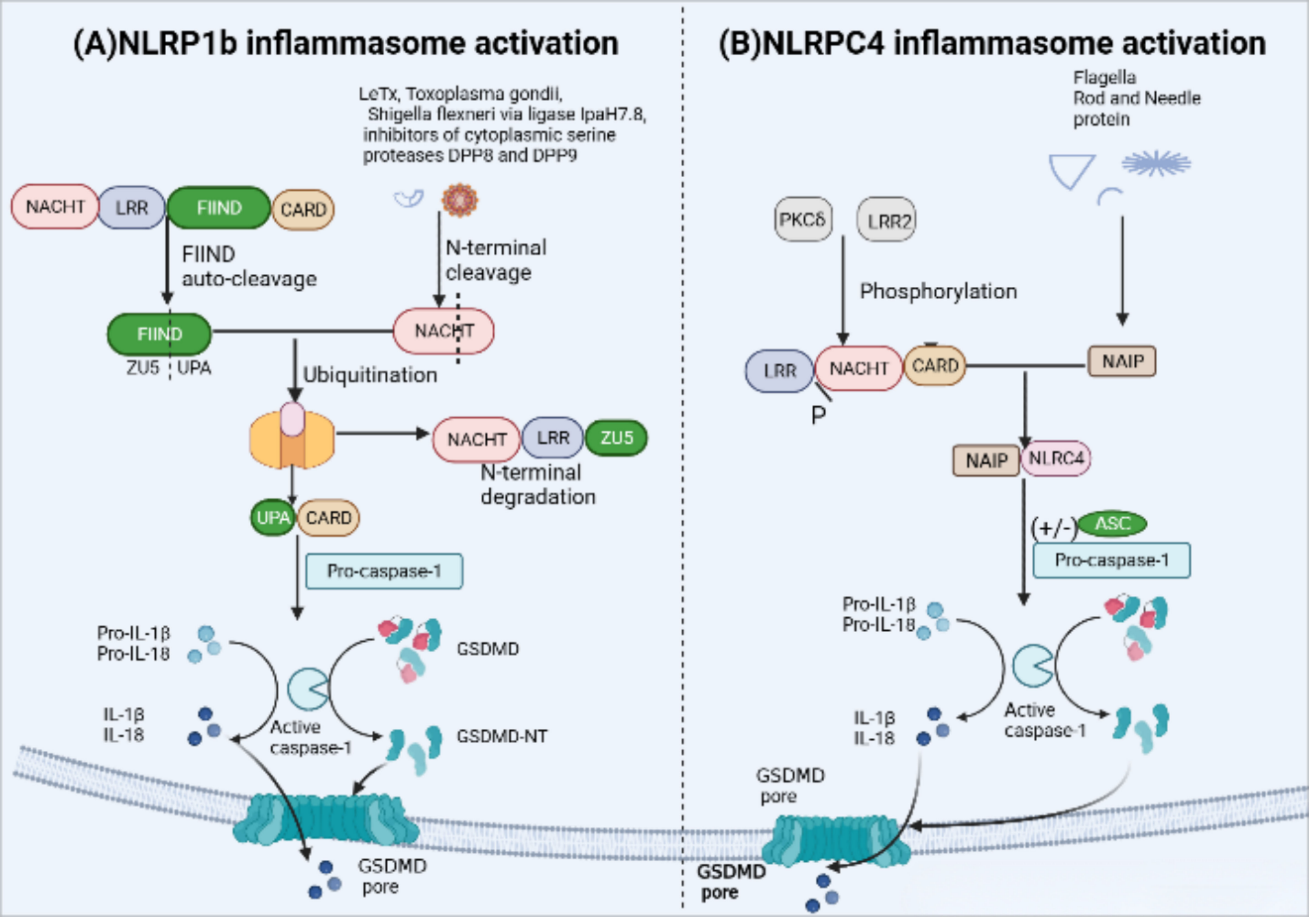
NLRP1b and NLRC4 inflammasome activation. **(A)** Activation of murine NLRP1b requires several steps of signal transmission. Firstly, the function-to-find domain (FIIND) containing a ZU5 subdomain and an UPA subdomain undergoes autoproteolytic cleavage, leading NLRP1b into two fragments in an autoinhibitory state until some specific stimuli, such asanthrax lethal toxin(LeTx), Toxoplasma gondii, IpaH7.8 inhibitors of DPP8 and DPP9, triggers inflammasome assembly. LeTx directly cleaves the N-terminal domain of the nucleotide-binding domain with ATPase activity(NACHT)–leucine-rich-repeat domain (LRR)–ZU5. Following cleavage, ubiquitination of NLRP1b marks it for proteasome degradation of the NACHT-LRR-ZU5 fragment and release of the active C-terminal fragment (UPA-CARD), which can initiate inflammasome assembly without ASC, leading to caspase-1-dependent release of IL-1b and IL-18 and pyroptosis. **(B)** The unique receptor NLR apoptosis inhibitory protein (NAIP) can detect bacterial flagellin, such as Salmonella typhi and Pseudomonas aeruginosa, and type III secretion system (T3SS) protein like the needle and rod protein components activation. Moreover, with or without ASC to form an inflammasome, phosphorylation of NLCR4 by PKCδ and leucine-rich repeat kinase 2 (LRRK2) triggers formation of an NAIP–NLRC4 complex and the recruitment of caspase-1.

Mice carry three NLRP1 analogues (NLRP1a-c) that lack the pyrin domain (66). The most representative NLRP1b is susceptible to Bacillus anthracis lethal toxin (LeTx), Toxoplasma gondii (69), IpaH7.8 secreted from Shigella flexneri ubiquitin ligase and inhibitors of cytoplasmic serine proteases DPP8 and DPP9 (70, 71). The FIIND domain contains a ZU5 subdomain and an UPA subdomain, whose auto-cleavage is required for NLPR1b inflammasome activation (72). Activation of NLRP1b via the N-end rule proteasomal degradation pathway represents a unified mechanism for its response to diverse activators (71, 73) (**Figure 4**). when NLRP1b is passed by LeTx, ubiquitination of NLRP1b induces proteasomal degradation of the NOD-LRR-ZU5 fragment, releasing the active UPA-CARD fragment that rapidly oligomerizes to engage downstream inflammasome effectors (74–76).

### 3.3 Activation mechanism of NLRC4 inflammasome

NLRC4 in human and mouse macrophages forms a NAIP-NLRC4 complex via the unique receptor NLR apoptosis inhibitory protein (NAIP) (77, 78), which is recognized by bacterial flagellin, such as Salmonella typhi and Pseudomonas aeruginosa, and type III secretion system (T3SS) protein like the needle and rod protein components activation (77, 79). In addition, in mouse macrophages, the NLCR4 inflammasome also requires PKCδ and leucine-rich repeat kinase 2 (LRRK2) phosphorylation to function (80, 81) (**Figure 4**). NLRC4 structurally contains an N-terminal CARD and lacks PYD, therefore, the NAIP-NLRC4 complex can directly recruit and activate pro-caspase-1 to form inflammasomes. However, the presence of ASC can significantly enhance NLRC4-mediated pyroptosis (44).

### 3.4 Activation mechanism of AIM2 inflammasome

**Figure 5:**
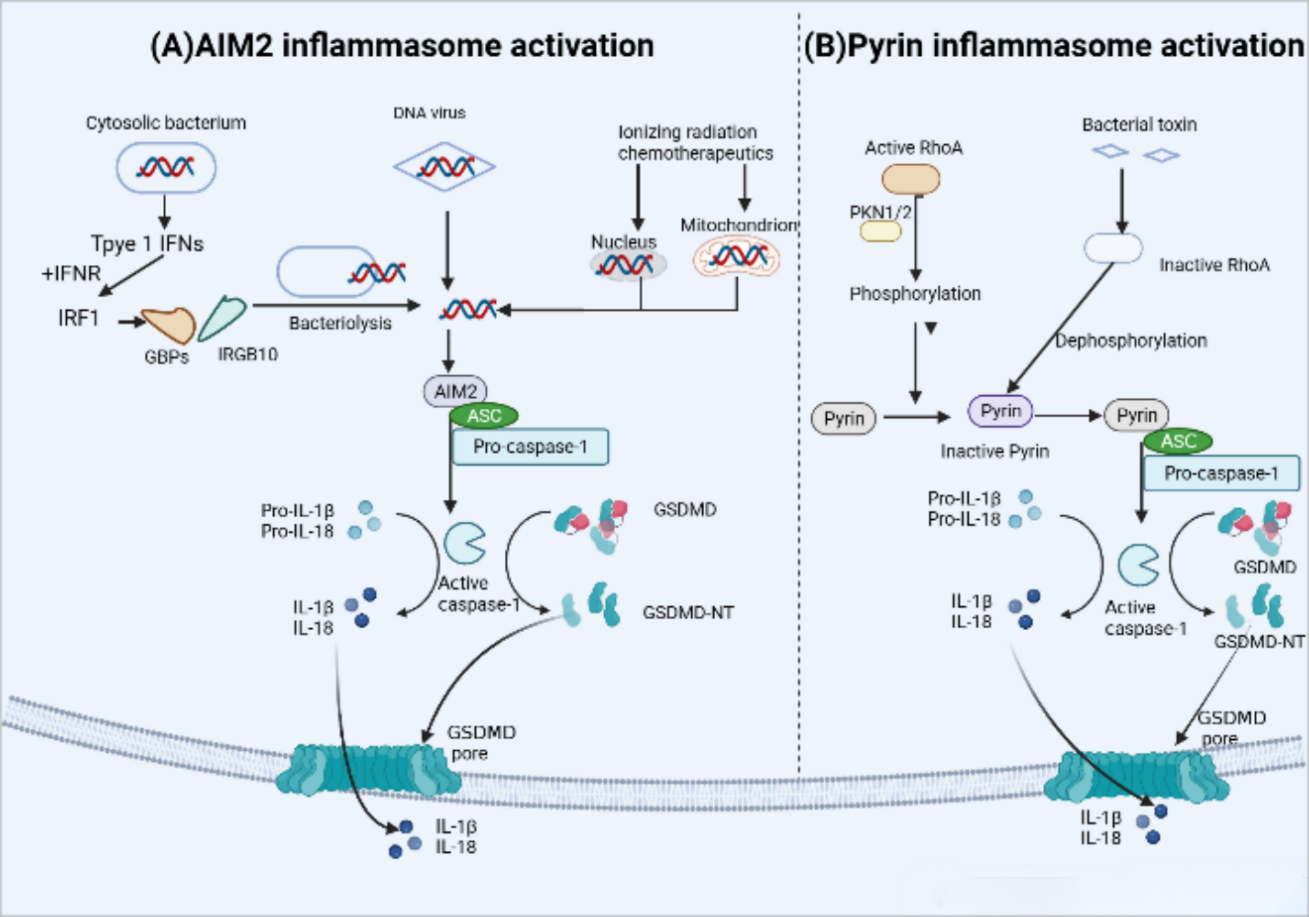
AIM2 and Pyrin inflammasome activation. **(A)** AIM2 inflammasome activation occurs in response to microbial or self-DNA. Cytosolic bacteria induce the production of type I IFNs which drive the expression of GBPs and IRGB10, targeting bacterial and vacuolar membranes for destruction. Bacterial DNA released into the cytoplasm to initiate AIM2 activation.In contrast, DNA viruses can activate AIM2 without type I IFN signaling. AIM2 also recognizes host cytoplasmic double-stranded DNA (dSDNA) leaked from nuclear or mitochondrial damage caused by Ionizing radiation chemotherapeutics. Upon binding to DNA, AIM2 participate in the following recruitment of ASC and pro-caspase-1,resulting in AIM2 inflammasome-caspase-1-mediated pyroptosis. **(B)** Under normal conditions, human and murine pyrin is phosphorylated by the Ras homolog family member A (RhoA) effector kinases, protein kinase N1/2 (PKN1/2) that keep pyrin in an inactive state. Bacterial toxins can inhibit RhoA activity and subsequent PKN1/2 phosphorylation, leading to the dephosphorylation of pyrin,which maintain it active status and recruits ASC and pro-caspase-1. Ultimately, active caspase-1 Inducts pyroptosis.

The AIM2 inflammasome in humans and mice is activated by microbial or self DNA (82) (**Figure 5**). Cytoplasmic bacteria induce the production of type I interferon (IFN), which leads to the collaboration of guanylate binding proteins (GBPs) and IRGB10, promoting the release of bacterial DNA into the cytoplasm and activating AM2 (83). On the other hand, DNA viruses such as mouse cytomegalovirus can activate AIM2 independently of type I IFN signaling (84). Additionally, the HIN-200 domain of AIM2 recognizes cytoplasmic double-stranded DNA (dSDNA) resulting from nuclear or mitochondrial damage, and then the pyrin domain recruits ASC and caspase-1 to form an active inflammasome complex (85, 86).

### 3.5 Activation mechanism of pyrin inflammasome

Mutations in the MEFV gene which encodes pyrin inflammasome (not to be confused with the pyrin domain), are associated with autoinflammation in familial Mediterranean fever (FMF). The pyrin inflammasome can be activated by virogenic toxins (**Figure 5**), such as the major virulence factor TcdB of Clostridium difficile, toxins of Vibrio parahaemolyticus (VopS), the ADP-ribosylating C3 toxin of Clostridium botulinum (87, 88). In the case of Clostridium difficile toxin TcdB, it inactivates the Rho GTPase, leading to the activation of the Pyrin inflammasome. Active RhoA, in turn, activates the protein kinase N1/2 (PKN1/2), which bind and phosphorylate pyrin inflammasome sensors, resulting in the inhibition of pyrin inflammasome activation (89).

## 4 Noncanonical pyroptotic pathway

The noncanonical pathway involves the intracellular protease cascade triggered by lipopolysaccharide (LPS) through two human homologs, caspase-4/5 (caspase-11 in mice). This cascade results in the processing and maturation of GSDMD, leading to the secretion of IL-1β and IL-18 (90, 91). In contrast to the typical inflammasome-mediated pyroptosis that requires various protein components for ligand sensing, assembly, and effector functions, caspase-4/5/11 possesses the ability to directly bind to cytoplasmic lipopolysaccharide (LPS) via its N-terminal CARD domain (92). It cleaves GSDMD, generating GSDMD-N, which translocates to the cell membrane and forms oligomers, creating membrane pores (93). It is important to note that the release of IL-1β and IL-18 occurs through secondary activation of the canonical pathway (17) (**Figure 2**). The noncanonical pathway-induced pore formation leads to potassium efflux, subsequently activating canonical pathway mediated by NLRP3 inflammasome and resulting in the secretion of IL-1β and IL-18 (94, 95). Furthermore, a recent study by Zhu et al. discovered a cytoplasmic lipopolysaccharide (LPS) sensor, an orphan nuclear receptor called Nur77, which binds to LPS and mitochondrial DNA and activates the atypical NLRP3 inflammasome (96). Extracellular LPS can enter the cytoplasm through endocytosis mediated by the Toll-like receptor 4(TLR4) or bacterial outer membrane vesicles (OMVs) (96–98). It is reported that in interferon γ-stimulated cells the host guanylate binding protein (GBP) facilitates the surface of Gram-negative bacteria into a multivalent signaling platform required for caspase-4 activation (99).

LPS-induced activation of noncanonical pathway triggers pyroptotic cell death and cytokine release through GSDMD pores, which is essential in the protection against cytoplasmic invading bacteria as well as in the induction of endotoxin shock. It is 1-palmitoyl-2-arachidonoyl-sn-glycero-3-phosphocholine (oxPAPC) and its derivatives that show potential as therapeutic targets for noncanonical inflammasomes during Gram-negative bacterial sepsis. OxPAPC, an oxidized phospholipid derived from the host, competitively binds to LPS and inhibits the activation of the noncanonical inflammasome by caspase-4/11 in macrophages (100). However, this inhibition is not observed in dendritic cells (101). Furthermore, Li et al. revealed the immune escape mechanism of Shigella flexneri, which evades pyroptosis mediated by the atypical inflammasome caspase-4/11 through the secretion of inhibitory effectors or modification of its LPS structure (102).

## 5 Gasdermins-dependent pyroptosis

**Figure 6:**
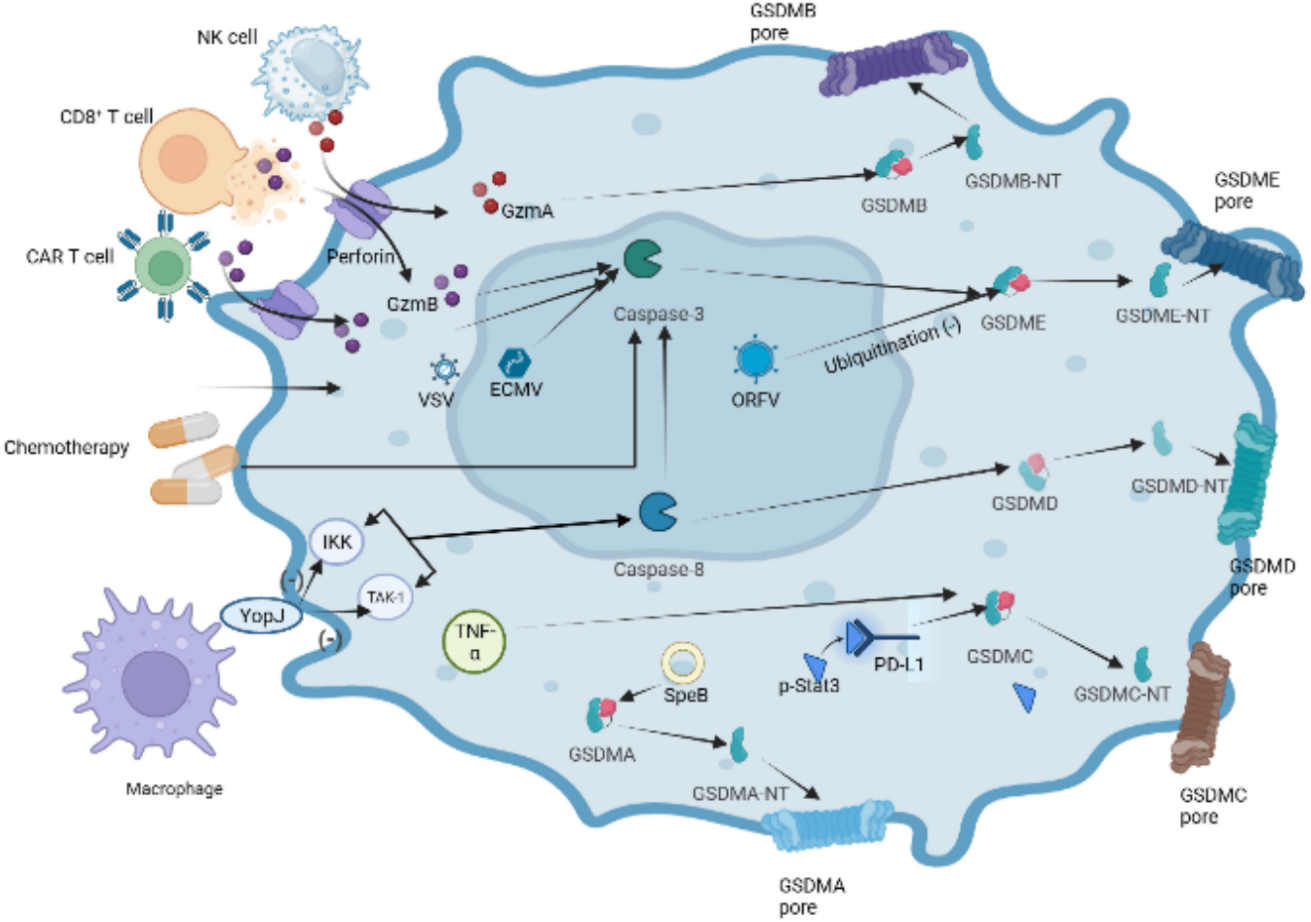
Other pathways of GSDMDs-dependent pyroptosis. In the granzyme-mediated pathway, GzmA secreted from NK cells and CD8+ T cells induces GSDMB-mediated pyroptosis. CAR-T cells can release a great deal of perforin and GzmB activating caspase-3,which cause GSDME cleaved by caspase-3 in target cells.Chemotherapeutic drugs could induce caspase-3-mediated GSDME cleavage with high GSDME expression and form GSDME-NT, causing pyroptosis of tumor cells. The inhibition of TAK1 and IKK activates caspase-8 via the lysosomal rag-ragulator complex, resulting in GSDMD cleavage and pyroptosis by Yersinia. Under hypoxia conditions, p-Stat3 interacts with PD-L1 and upregulates the transcription of GSDMC. Activated by TNF-α, caspase-8 cleaves GSDMC into GSDMC-NT, resulting in pyroptosis. Group A Streptococcal (GAS) cysteine protease SpeB cleaves directly to induce GSDMA-dependent pyroptosis.

In recent years, there have been significant advancements in the understanding of pyroptotic underlying mechanisms, leading to the revision and redefinition of pyroptosis. Studies have increasingly reported that pyroptosis can occur without the activation of inflammasome and inflammatory caspases (103, 104). Instead, this cell death process relies on members of the gasdermin family. Some studies have proposed redefining “pyroptosis” as “call necrotic death induced by gasdermins” to better capture its characters of dependence on gasdermin family proteins (93, 105). The gasdermin protein family comprises six members: gasdermins A-E and deafness autosomal recessive type 59 (DFNB59 or pejvakin). Except for DFNB59, all other gasdermins have been implicated in the induction of pyroptosis, as demonstrated in studies (35, 106). In the following sections, we will provide a brief overview of some research findings on the involvement of the five GSDMs in pyroptosis (**Figure 6**).

### 5.1 GSDMA

GSDMA, identified as a pyroptosis-triggering effector, whose activator bypasses inflammasomes and caspases has been reported recently (104, 107). GSDMA undergoes cleavage by the group A Streptococcal (GAS) cysteine protease SpeB, leading to the formation of pores in the cell membrane. This finding provide a piece of evidence that GSDMs can function as direct sensors of exogenous proteases, independent of host inflammasome sensors.

### 5.2 GSDMB

GSDMB plays a role in promoting the cleavage of GSDMD by enhancing the enzymatic activity of caspase-4, thereby participating in non-canonical pyroptosis (108). In 2020, it was found that lymphocyte-derived GzmA cleaves GSDMB, leading to pyroptosis in natural killer cells and cytotoxic T lymphocytes, indicating the activation of non-caspase factors (105). Similarly, GSDMB has recently been discovered to possess direct bactericidal activity through its pore-forming capability (109). However, compared with other gasdermin s, the pyroptosis function of GSDMB has been questioned by some researchers (108, 109). Recently, Zhong et al. discovered the Shigella flexneri ubiquitin-ligase virulence factor IpaH7.8 can degrade GSDMB through a similar mechanism as target human GSDMD. Author considered the structure of GSDMB suggests stronger autoinhibition than other gasdermins, which is related to relatively poor pyroptosis function of GSDMB (110).

### 5.3 GSDMC

Caspase-8 has been reported to specifically cleave GSDMC to induce tumor cell scorch death (27, 111). Under hypoxia conditions, p-Stat3 physically interacts with programmed cell death ligand-1 (PD-L1), leading to its nuclear translocation and upregulation of GSDMC gene expression. Caspase-8 is specifically processed by macrophage-derived TNF-α to cleave GSDMC to induce scorch death (27). The function of PD-L1 beyond immune checkpoint regulation has been confirmed by these studies.

### 5.4 GSDMD

The innate immune system exhibits a complex interplay between cell death pathways and caspase-8 is extensively involved in regulating pathways of apoptosis (112, 113). Studies have shown that Yersinia-infected mouse macrophages can inhibite kinase-1 (TAK1) or IκB kinase (IKK) through the effector protein YopJ, which results in caspase-8-dependent cleavage of GSDMD, subsequently triggering GSDMD-mediated pyroptosis (114, 115). Recently, it was demonstrated that the lysosomal membrane-anchored follicle protein (FlCN)-folliculin-interacting protein 2 (FNIP2)-Rag-Ragulator complex, which can recruite Fas-Associated protein with Death Domain (FADD), receptor-interacting serine/threonine-protein kinase 1 (RIPK1) and caspase-8 and result in RIPK1 phosphorylation and caspase-8 activation, is necessary for RIPK1-caspase-8-dependent GSDMD-mediated pyroptosis (116).

### 5.5 GSDME

Active caspase-3 cleaves GSDME to generate an N-terminal fragment of GSDME and induces pyroptosis. Chemotherapeutic drugs can trigger caspase-3-gasdermin E mediated pyroptosis in tumor cells (117, 118). Interestingly, chemotherapeutic drugs induce caspase-3 mediated apoptosis in cells with low expression of GSDME, which indicates complex interactions between cell death Pathway in the immune system. In addition, recent reports have demonstrated that GSDME is cleaved by caspase-3 activated by granzyme B (GZMB) (119). GZMB, a serine protein highly expressed in cytotoxic lymphocytes, can augment the immune response of NK cells and CD8+ T cells. Chimeric antigen receptor (CAR) T cells can release more granzyme B which triggers the cleavage of GSDME to mediate B leukemic cells pyroptosis, and perforin than nontransduced natural T cells (119) (**Figure 6**). Several viruses have been found to induce pyroptosis through GSDME. For instance, Oncolytic parapoxvirus (ORFV) stabilizes GSDME by reducing its ubiquitination, leading to tumor cell pyroptosis (120). Additionally, infection with vesicular stomatitis virus (VSV) or encephalomyocarditis virus (ECMV) has been shown to trigger caspase-3/GSDME mediated pyroptosis in bone marrow-derived macrophages (BMDM) (118).

## 6 The relationship between pyroptosis and endometrial cancer

### 6.1 Inflammasome in endometrial cancer

The NLRP3 inflammasome is closely associated with the progression of various gynecological diseases., including polycystic ovary syndrome (121), cervical cancer (122, 123), ovarian cancer and endometriosis (EM) (124). EM is an inflammation-dependent disease with adhesive and invasive properties, sharing similarities with malignancies. NLRP3 inflammasome levels are relatively low in normal endometrium but significantly upregulated in EM and EC tissues (124–126). Zhou et al. demonstrated that NLRP3 activation in macrophages increased the secretion of IL-1β, promoting the migration and invasion capabilities of endometrial stromal cells (ESCs) *in vitro*. Murakami et al. administered the NLRP3 inhibitor MCC950 to ovarian endometriosis (OE) mice and showed that inhibition of NLRP3 inflammasome activity reduced the expression of IL-1β and Ki67 in cyst-derived stromal cells (CSCs), leading to a significant inhibition of OE lesion size (127).

Estrogen receptors α (ERα) and ERβ play a crucial role in the development and malignant progression of type 1 endometrial cancer (128). 17β-estradiol (E2) binds to ERα and activates fibroblasts and myofibroblast cells, leading to the secretion of cell cycle proteins (MAD2L1, CDKN1A, and CEBPβ) and growth factors (IGF and TGF), which promotes epithelial-mesenchymal transition (EMT) and alters the expression levels of cell adhesion proteins, such as E-cadherin and β-catenin, enhancing resistance to apoptosis and promoting the activity of migration, and invasion (129). ERβ, which is less studied, is considered a gynecological tumor suppressor and plays an opposing role to ERα in EC development (130). Moreover, It can impair the transcriptional function of ERα (131). A study in 2019 found that E2 acts through ERβ, enhances the activation of NLRP3 inflammasome, and promotes the progression of endometrial cancer (132). These findings suggest that the abnormally expressed NLRP3 inflammasome in EC could be a specific target for clinical therapy. However, further research and exploration are needed to understand the impact of pyroptosis involving NLRP1, NLRC4, AIM2, and PYRIN inflammasome on the biological behavior of EC.

### 6.2 Gasdermins in endometrial cancer

GSDMD, GSDME, and GSDMB are the main pyroptosis-related GSDMs expressed in the endometrium (93). Yang et al. discovered that the expression of GSDMD was higher in EC and atypical hyperplastic endometrial tissues compared to benign endometrial tissues (126). Moreover, GSDMD serves as a prognostic marker and potential therapeutic target for endometrial cancer, considering GSDMD related to Wnt signaling and substance metabolism pathways.

Deafness autosomal dominant 5 (DFNA5), a potential tumor suppressor gene, has gained significant attention from researchers in recent years. Hu et al. discovered that DFNA5 expression was significantly lower in kidney chromosome (KICH), prostate adenocarcinoma (PRAD) and breast invasive carcinoma (BRCA) compared to adjacent normal tissues (133). In addition, methylation of the GSDME gene promoters in breast cancer has been associated with poor 5-year survival rate (134, 135). Several studies have reported downregulation of the GSDME gene in EC cells compared to surrounding normal tissues (133, 136). However,tumor microenvironment (TME) with low GSDME expression has been found to have reduced tumor-infiltrating lymphocytes (TIL, CD8+ T and NK), GzmB, and perforin (PFN), potentially leading to an immunotherapy-unfavorable microenvironment in EC (137). The mechanism by which GSDMB affects EC is currently unclear, and there is limited research on the role of GSDMB in EC. Pyroptosis mediated by GSDMs is emerging as a novel antitumor therapy (119), and further studies are needed to elucidate the mechanistic role of GSDMs in EC.

### 6.3 Dual roles of pyroptosis in endometrial cancer

Tumor cells, stromal cells, immune cells, and extracellular matrix (ECM) components in the tumor microenvironment (TME) form a complex network (138), which collectively regulates tumor cell proliferation and metastasis under hypoxic and inflammatory conditions. Pyroptosis plays a dual role in the occurrence and progression of EC. On one hand, the inflammatory response amplified by pyroptosis promotes the formation of an inflammatory microenvironment conducive to the growth of EC cells. On the other hand, inducing pyroptosis may inhibit the malignant progression of EC (**Table 2**).

**Table 2:** The regulatory role of pyroptosis-related targets in endometrial cancer progression.

Target	Mechanism	Function	Reference
E2	Activates ERβ-NLRP3-caspase-1-GSDMD	Induce pyroptosis and promote endometrial cancer	(132)
HKDC1	Activates ROS-NLRP3-caspase-1-GSDMD	Induce pyroptosis and promote endometrial cancer	(125)
Hydrogen	Activates ROS-NLRP3-caspase-1-GSDMD	Induce pyroptosis and suppress endometrial cancer	(126)

#### 6.3.1 Pyroptosis-mediated inflammatory microenvironment promotes endometrial cancer progression

Numerous cohort studies and meta-analyses have confirmed a positive correlation between obesity, insulin resistance, diabetes, and endometrial cancer (139–142). This association is linked to systemic or endometrium local chronic inflammatory conditions of obese and diabetic patients (143, 144). The imbalance of adipokines and cytokines in obese women creates TME conducive to the development and progression of endometrial cancer. Obese individuals exhibit reduced secretion of the anti-inflammatory adipokine adiponectin and increased secretion of inflammatory adipokines, such as leptin, which promote EC proliferation and metastasis. Inflammatory cells in adipose tissue show elevated secretion of IL-6, IL-11 and tumor necrosis factor-α (TNF-α). Poor blood glucose regulation in diabetic patients is often associated with increased levels of IL-1β and TNF-α (58).*In vitro* studies have demonstrated the ability of IL-1β to promote the proliferation and metastasis of human endometrial stromal cells (HESCs) (145).

Pyroptosis, as a pro-inflammatory form of cell death, is characterized by the secretion of IL-1β and IL-18. Its role in diabetes mellitus (DM)-associated EC has attracted scholarly attention. Guo et al. discovered that under hyperglycemia (HG) conditions, hexokinase domain protein 1 (HKDC1) activates canonical NLRP3 inflammasome-mediated pyroptosis through mitochondrial ROS. This process creates a pro-inflammatory and acidic TME that promotes the malignant progression of EC (125). *In vitro* experiments further demonstrated that lncRNA-HOXC-AS2 acts as a competing endogenous RNA (ceRNA) to potentially inhibit miR-876-5p, resulting in the upregulation of HKDC1. These findings reveal the potential therapeutic significance of targeting the lncRNA-HOXC-AS2/miR-876-5p/HKDC1 axis as a treatment approach for HG-associated EC (125).

#### 6.3.2 Inducing pyroptosis of EC cells to exert anti-endometrial cancer effect

In recent years, the regulation of tumor cell pyroptosis as an anti-endometrial cancer treatment strategy has gained confirmation. Yang et al. confirmed through *in vivo* and *in vitro* experiments that molecular hydrogen can activate ROS and mtROS production in endometrial cancer cells. This activation triggers the ROS-NLRP3-caspase-1-GSDMD pathway, leading to pyroptosis and influencing the biological behavior of endometrial cancer cells. In mouse models transplanted with EC tissue, this intervention resulted in reduced tumor volume and weight (126). Hydrogen molecules, known as natural antioxidants, selectively reduce hydroxyl radicals, the most cytotoxic ROS, without affecting other physiological ROS (146). The anti-oxidative stress, anti-inflammatory, and anti-apoptotic effects of hydrogen have been confirmed in animal models of cardiovascular and cerebrovascular diseases, chronic liver disease, pancreatitis, and other conditions (146–148). In clinical trials, hydrogen inhalation has shown the ability to reduce airway inflammation in patients with asthma and COPD (149).

In a hypoxic environment, tumour cells activate hypoxia-inducible factor 1α (HIF-1α) to reprogramme their energy metabolism pathways to maintain high proliferation rates and promote tumour growth, invasion and neointima formation. Studies have shown that over expression of HIF-1α in EC promotes lymph node metastasis and myometrial invasion of tumour cells, which is significantly associated with poor prognosis of EC (150). It is known that hypoxia up-regulates cyclin-dependent kinase inhibitors p27 and p53, causing cell cycle arrest and thus favoring the survival of endometrial cancer cells (151, 152). Notably it has recently been proposed that high expression of HIF-1α in EC tissues also enhances the anti-pyroptosis characteristics of tumour cells (153). However, this hypothesis awaits further scientific proof in the form of experiments.

## 7 Prospects of pyroptosis in anti-endometrial cancer therapy

Recent studies have demonstrated the feasibility and clinical potential of using pyroptosis as an anticancer therapy mechanism. Many researchers are attempting to treat cancer by modulating pyroptosis with other oncology treatments to inhibit tumor cell proliferation, migration and invasion.

### 7.1 Radiotherapy

Postoperative radiation therapy (RT), comprising total pelvic external beam radiation therapy (EBRT) and intracavity vaginal brachytherapy (VBT), is a crucial adjuvant treatment for women with intermediate-risk and high-risk EC. It effectively decreases the risk of local EC recurrence (154, 155). The challenge in radiotherapy for human tumors has always been to achieve maximal tumor cell eradication while minimizing radiotoxicity to adjacent normal organs.

Currently, pyroptosis has been implicated in radiation-induced damage to normal tissues. For instance, Li et al. demonstrated that high-dose X-ray irradiation triggered pyroptosis in human umbilical vein endothelial cells (HUVECs) (156). The activation of caspase-1 and release of IL-1β are influenced by Panx1, an important factor in pyroptosis (157). Hu et al. reported that ionizing radiation and chemotherapeutics-induced DNA damage in the nucleus can activate the AIM2 inflammasome, leading to caspase-1-mediated pyroptosis in intestinal epithelial cells and myeloid cells (85). Hence, targeting the AIM2 inflammasome could potentially alleviate myelodysplastic syndrome or gastrointestinal toxicity in cancer patients undergoing radiation or chemotherapy. Wu et al. found that radiation-induced pyroptosis in small intestinal cells involves the activation of the NLRP3 inflammasome/caspase-1 pathway through ROS generation (158). Notably, FlaAN/C, a flagellin derivative, was shown to inhibit radiation-induced ROS production and protect small intestinal tissue (158). Although the clinical application of pyroptosis blockade to mitigate the toxic side effects of radiotherapy requires extensive preclinical validation, these findings offer valuable insights into the mechanisms underlying radiation-induced cytotoxicity.

### 7.2 Chemotherapy

Cancer chemotherapy is a rapidly evolving field faced with challenges of drug resistance and adverse reactions. Precise regulation of pyroptosis has emerged as a potential solution to address tumor chemotherapy resistance and adverse events (AEs) (117, 159). Platinum- and paclitaxel-based chemotherapy resistance and toxic side effects pose significant challenges in the treatment of advanced, recurrent and metastatic EC (160). A multicenter retrospective cohort study analyzing 262 patients with recurrent endometrial cancer who had a history of receiving first-line platinum-based chemotherapy, found the response rates at second-line chemotherapy for patients with platinum-free intervals of <6 months, 6-11 months, 12-23 months and ≥24 months were 25%, 38%, 61% and 65%, respectively (161). It has been demonstrated that a ruthenium(II) polypyridine complex, Δ-Ru1, in combination with paclitaxel, can enhance the anticancer effect on paclitaxel-resistant cancer cells through caspase-1/GSDMD-mediated pyroptosis (162). Differential expression of GSDME in tumor cells versus normal cells has been associated with chemotherapy sensitivity and AE (137). Cancer cells with low GSDME expression undergo caspase-3-mediated apoptosis upon chemotherapy, while those with high GSDME expression shift towards pyroptosis (117). Decitabine and azacitidine have been shown to reverse GSDME silencing, increasing tumor cell sensitivity to chemotherapeutic drugs (117). These drugs are used in the treatment of myelodysplastic syndrome (MDS) or acute myeloid leukemia (AML) (163, 164). Compared with GSDME-/- mice, wild-type mice exhibited increased severity of cisplatin-induced weight loss and spleen size reduction. Meanwhile, intraperitoneal injection of 5-FU exacerbated intestinal bleeding, inflammatory cell infiltration and crypt loss in wild-type mice (117). Reversing the expression level of GSDME in cancer cells may hold the key to leveraging pyroptosis therapy for EC treatment in the coming years.

In recent years, pyroptosis has emerged as a distinct form of cell death that can be triggered by exogenous drugs in various cancers, offering a unique anti-tumor mechanism different from apoptosis or autophagy. For instance, cisplatin and paclitaxel have been shown to induce pyroptosis in lung cancer A549 cells through caspase-3/GSDME activation (165). Lobaplatin was proven to induces ROS/JNK (c-Jun N-terminal kinase) signalling to induce the pyroptosis via a novel Bax-caspase-GSDME pathway in colon cancer cells (166). Similarly, 2-(α-naphthoyl) ethyltrimethylammonium iodide (α-NETA) was found to inhibit epithelialization and induce caspase-4-triggered pyroptosis in epithelial ovarian cancer, restraining the proliferation of it (167). Tanshinone IIA has demonstrated anticancer activity against cervical cancer cells by upregulating miR-145/GSDMD signaling (168). The underlying mechanisms of these compounds regulating pyroptosis pathway are summarized in **Table 3**.

**Table 3:** Compounds inducing pyroptosis signal pathways in cancers.

Classifcation	Compounds	Cancer types	Mechanisms of pyroptosis induction	References
Chemotherapy drugs	Decitabine/Azacitidine	MDS/AML	Caspase3/GSDME	(117)
	Topotecan/Etoposide/Cisplatin	neuroblastoma	Caspase3/GSDME	(117)
	Cisplatin/PTX	Lung cancer	Caspase-3/GSDME	(165)
	Lobaplatin	CRC	ROS/JNK/Caspase-3/GSDME	(166)
	DOX	breast cancer	ROS/JNK/Caspase-3/GSDME	(159)
Natural products	Dioscin	OS	Caspase-3/GSDME	(169)
	DHA	ESCC	PKM2-Caspase-8/3-GSDME	(170)
	CME	Lung cancer	Caspase-3/GSDME	(170, 171)
	CBD	HCC	Caspase-3/GSDME	(25)
	Tanshinone IIA	Cervical cancer	miR-145/Caspase-1/GSDMD	(168)
	Berberine	HCC	Caspase-1	(172)
Reagents	C10	Prostate cancer	PKCδ/JNK/Caspase-3/GSDME	(173)
	BI 2536	OC	Caspase-3/GSDME	(28)
	α-NETA	OC	Caspase-4/GSDMD	(167)
	13d	Lung Cancer	inhibit NF-kB pathway	(174)

PTX, paclitaxel; DOX, doxorubicin; DHA, Dihydroartemisinin; CME, Cordyceps militaris extract. CBD, Cannabidiol; C10, 3’,5’-diprenylated chalcone; α-NETA, 2-(α-naphthoyl) ethyltrimethylammonium iodide; 13d, low toxicity NF-kB inhibitor; MDS, myelodysplastic syndromes; AML, acute myeloid leukemia; CRC, colorectal cancer; OS, osteosarcoma; ESCC,esophageal squamous cell carcinoma; HCC, hepatocellular carcinoma; OC, ovarian cancer; GSDME, gasdermin E; GSDMD, gasdermin D; ROS, reactive oxygen species; JNK, c-Jun N-terminal kinase; PKM2, pyruvate kinase isoform M2; miR-145, microRNA-145; PKCδ, protein kinase C delta; NF-kB, Nuclear factor kB.

### 7.3 Targeted therapy

Since the identification of 4 distinct EC subgroups associated with differential survival by The Cancer Genome Atlas (TCGA), clinical trials investigating targeted therapies for EC have been expanding and showing promise. For instance, the combination of TC (carboplatin and paclitaxel) plus trastuzumab has demonstrated increased progression-free survival (PFS) (HR = 0.46; 90% CI, 0.28-0.76) and overall survival (OS) (HR=0.58; 90% CI, 0.34–0.99) in patients with advanced or recurrent uterine-serous-carcinoma (USC) exhibiting human epidermal growth factor receptor 2 (HER2) overexpression (175). Vascular endothelial growth factor (VEGF) is considered a crucial factor in tumor angiogenesis, regulating tumor proliferation, invasion, migration, and neovascularization (176–178). Combining anti-angiogenic drugs, such as bevacizumab, with carboplatin and paclitaxel has significantly prolonged OS of EC (OS HR=0.28; 95% CI, 0.14-0.59), particularly those with TP53 mutations, compared to paclitaxel and carboplatin regimens (179, 180). Lenvatinib combined with pembrolizumab has shown significant improvements in PFS and OS for EC patients and has received FDA approval for second-line treatment of recurrent or metastatic endometrial cancer (181). However, lenvatinib is associated with common adverse reactions in clinical applications, including fatigue, fever, nausea, vomiting, hypertension, diarrhea, and more severe events like liver injury and intracranial hemorrhage (182, 183). Grade 3 or higher AEs often impact the tolerability of recurrent EC patients.

Anti-angiogenic drugs exhibit anti-tumor effects by triggering pyroptosis, which is associated with an amplified inflammatory response leading to adverse drug events. Sorafenib, a broad-spectrum kinase inhibitor used for hepatocellular carcinoma (HCC) treatment, induces macrophage pyroptosis by activating caspase 1. The released cytokines synergistically enhance NK cell effector functions, resulting in effective tumor cell killing (184). Lenvatinib, on the other hand, induces ROS-caspase 3-GSDME-dependent pyroptosis in GSDME-expressing cells through the loss of mitochondrial membrane potential (185). However, ZLF-095, a novel VEGFR inhibitor, effectively inhibits liver and colorectal cancer proliferation without inducing pyroptosis, thus reducing the adverse effects associated with lenvatinib (185). This study highlights the potential association between pyroptotic amplified inflammatory response and adverse reactions caused by systemic treatment of lenvatinib. In the treatment of anaplastic thyroid cancer (ATC), the combination of apatinib, an anti-angiogenic targeted drug, with melittin activates caspase-1/GSDMD and caspase-3/GSDME-mediated pyroptosis. This combination reduces the incidence of adverse events by decreasing the therapeutic dose of apatinib alone. Furthermore, positive feedback interaction was observed between the two GSDM-mediated pyroptotic pathways (186). Currently, there is limited research on whether anti-angiogenic drugs activate pyroptosis in EC to exert anti-tumor effects. Understanding the biological effects of pyroptosis factors in EC targeted therapy is crucial for optimizing treatment strategies and improving prognosis in the future.

### 7.4 Immunotherapy

Pyroptosis plays a crucial role in converting “cold” tumors into “hot” ones and exhibits a synergistic effect when combined with immune checkpoint blockade (ICB) therapy. The exploration and analysis of molecular subtypes have become significant aspects of anti-tumor drug research in EC. While Immune checkpoint inhibitor shows positive responses in patients with microsatellite instability-high (MSI-H) and mismatch repair deficient (MMRd) EC (187, 188), the overall response rate remains low due to ineffective infiltration or activation of T lymphocytes and NK cells within the tumor microenvironment. Notably, despite being of the mismatch repair deficient (MMRd) subtype, 22% of the cases still exhibit low levels of TIL-Low, with such matters being more prevalent in p53 abnormal type (p53abn) and p53 wild type (p53wt) (189).

However, GSDME expression can modulate the quantity and function of tumor-infiltrating natural killer (NK) cells and CD8+ T lymphocytes, which triggers cytotoxic granule-mediated PFN-dependent killing and cytokine secretion, thereby enhancing the patients’ anti-tumor immunity (137, 190). Cancer cells have developed two strategies to evade GSDME-mediated tumor suppression: epigenetic suppression and mutation of GSDME (135). Consequently, upregulating GSDME expression in EC, potentially through the use of DNA methylation inhibitors, is expected to improve the response rates to ICB therapy of all molecular subtypes.

In addition, statins have been reported to enhance antitumor immunity by promoting caspase-1/GSDMD-induced pyroptosis and synergistically inhibiting ARID1A-mutated clear cell ovarian cancer (OCCC) when combined with ICB therapy (191). These findings provide valuable insights for the development of drugs targeting pyroptosis-related pathways to activate anti-tumor immunity in endometrial cancer.

## 8 Pyroptosis in diagnosis and prognosis of endometrial cancer

Molecular typing of EC has become widely used for prognostic assessment and treatment guidance. Currently, the activation of signaling pathways including PI3K/Akt, P53, mitogen-activated protein kinase, and Wnt/β-catenin is known to be closely associated with the pathogenesis of EC (10). Mutations in genes such as PTEN, PI3KCA, POLE, CTNNB1, and TP53, loss of DNA mismatch repair proteins, expression of estrogen receptors and progesterone receptors, and overexpression of HER2 are involved in the diagnosis and prognosis of EC (6, 10). Additionally, several studies have explored the expression levels of pyroptosis-related genes and proteins in EC patients, along with their correlation with patient prognosis, through bioinformatics analysis (**Table 4**). These studies aim to identify more effective therapeutic targets and diagnostic biomarkers associated with prognosis.

**Table 4:** Pyroptosis-related genes in patients with endometrial cancer.

Related genes	Diagnostic potential	Prognostic potential	Reference
CASP3, GPX4, GSDMD, NOD2, PYCARD and TIRAP		√	(192)
BAK1, CHMP2A, GSDMD, IRF2, GPX4, GSDMB, TIRAP and TNF		√	(193)
HM13-IT1, FIRRE, NNT-AS1, ATP6V0E2-AS1, AL353622.1 and POC1B-AS1		√	(194)
NLRP1, NLRP6, TNF, NOD2, IL18, ELANE, CASP5, NLRP3, AIM2 and IL-6		√	(195)
NFKB1, EEF2K, CTSV, MDM2, GZMB, PANX1 and PTEN		√	(196)
AC087491.1, AL353622.1, AL035530.2, LINC02036, AL021578.1, AL390195.2, AC009097.2, AC004585.1 andAC244517.7		√	(197)

## 9 Discussion and outlook

The understanding of the regulatory mechanisms and key regulators of pyroptosis, an emerging form of cell death, has rapidly advanced since its discovery. The precise regulation of pyroptosis in EC cells *in vivo* may offer a new treatment strategy for those patients who are ineligible for surgery, radiotherapy or chemotherapy. Pyroptosis, as a proinflammatory form of cell death, exhibits a dual role in the occurrence and development of EC. On one hand, the inflammatory environment induced by pyroptosis can promote the growth of EC cells and contribute to tumor formation, invasion, and metastasis. On the other hand, the induction of pyroptosis in EC cells can exert anti-tumor activity. This dual role, possibly attributed to the complexity of the pyroptosis pathway and its components, poses significant challenges for the clinical regulation of EC cell pyroptosis as a treatment. Numerous studies have demonstrated that conventional tumor treatments not only induce cancer cells apoptosis, but also trigger pyroptosis. It seems feasible and promising that combining pyroptosis with other tumor treatments, such as radiotherapy, chemotherapy and immunotherapy, to treat EC by regulating pyroptosis to suppress the proliferation, migration, and invasion of tumor cells.

This article provides a comprehensive review of physiological and pathological effects as well as the molecular mechanism of pyroptosis, with a specific focus on its research progress in the development, prognosis, and treatment of EC. However, these studies represent only a fraction of the research conducted thus far. Pyroptosis is expected to play a central role in EC therapy in the coming years. For instance, precise regulation of EC pyroptosis to directly exert anti-tumour activity, in addition, induction of EC pyroptosis can increase the response rates of first-line chemotherapy and immunotherapy for advanced and recurrent EC, and lessen the AEs of radiotherapy, chemotherapy and targeted therapy. Currently, numerous unanswered questions remain. EC is characterized by highly heterogeneous and it is not clear how sensitive different molecular subtypes of EC are to pyroptosis. Additionally, it is important to explore the regulators and mechanisms that influence the sensitivity of EC cells to pyroptosis. Furthermore, apart from NLRP3 inflammasome activation, there may be other pathways that induce pyroptosis and trigger distinct biological effects. The functional implications of different gasdermins and, in particular, the roles of GSDMB and GSDME in EC remain largely unknown. Addressing these issues will require further comprehensive investigations through massive preclinical and clinical studies.

## Author contributions

TP: Writing – original draft. CZ: Writing – original draft. WC: Writing-final draft. XZ: Writing – review & editing. WW: Writing – review & editing. WY: Writing – review & editing. RL: Writing – review & editing.

## Glossary

BMI: body mass index
FIGO: The International Federation of Gynecology and Obstetrics
TCGA: The Cancer Genome Atlas
OS: overall survival
GSDMs: gasdermins
GSDMs-CT: gasdermins-N terminal
GSDMs-NT: gasdermins-C terminal
IL-1β: interleukin-1β
IL-18: interleukin-18
ATP: adenosine triphosphate
HMGB1: high mobility group protein box 1
RCD: regulated cell death
ACD: accidental cell death
PCD: programmed cell death
GSDMD: gasdermin D
GzmB: Granzyme B
GzmA: Granzyme A
TME: tumor microenvironment
ASC: apoptosis-associated speck-like protein
PRRs: pattern recognition receptors
NOD: nucleotide-binding oligomerization domain
LRR: leucine-rich repeat
AIM2: absent in melanoma 2
PYD: pyrin domain
CARD: recruitment domain
PAMPs: pathogen-associated molecular patterns
DAMPs: danger-associated molecular patterns
NACHT: oligomerization domain with ATPase activity
mtROS: mitochondrial reactive oxygen species
Ox-mtDNA: oxidized mitochondrial DNA
TGN: trans-Golgi apparatus
dTGN: disassembled trans-Golgi network structure
PI4P: phosphatidylinositol 4-phosphate
EECS: endoplasmic reticulum-endosome membrane contact sites
HRV: human rhinovirus
UVB: Ultraviolet B
DPP8: dipeptidyl peptidase 8
DPP9: dipeptidyl peptidase 9
LeTx: lethal toxin
NAIP: NLR apoptosis inhibitory protein
LRRK2: leucine-rich repeat kinase 2
PKCδ: protein kinase Cδ
IFN: type I interferon
GBPs: guanylate binding proteins
IRGB10
FMF: Mediterranean fever
PKN1: protein kinase N1
PKN2: protein kinase N2
oxPAPC: 1-palmitoyl-2-arachidonoyl-sn-glycero-3-phosphocholine
DFNB59 or pejvakin: deafness autosomal recessive type 59
GAS: group A Streptococcal
PD-L1: Programmed cell death ligand 1
FlCN: follicle protein
FNIP2: folliculin-interacting protein 2
FADD: Fas-Associated protein with Death Domain
RIPK1: receptor-interacting serine/threonine-protein kinase 1
CAR-T: Chimeric antigen receptor T
ORFV: Oncolytic parapoxvirus
ECMV: encephalomyocarditis virus
NK: natural killer
BMDM: bone marrow-derived macrophages
EM: endometriosis
ESCs: endometrial stromal cells
OE: ovarian endometriosis
CSCs: cyst-derived stromal cells
ERα: estrogen receptors α
ERβ: estrogen receptors α
E2: 17β-estradiol
MAD2L1: mitotic arrest deficient 2 like 1
CDKN1A: Cyclin-Dependent Kinase Inhibitor 1A
CEBPβ: CCAAT enhancer binding protein beta
IGF: Insulin-Like Growth Factor
TGF: transforming growth factor
EMT: epithelial-mesenchymal transition
DFNA5: Deafness autosomal dominant 5
BRCA: breast invasive carcinoma
KICH: kidney chromosome
IL: tumor-infiltrating lymphocytes
TME: tumor microenvironment
PFN: perforin
ECM: extracellular matrix
TNF-α: tumor necrosis factor-α
HESCs: human endometrial stromal cells
DM: diabetes mellitus
HG: hyperglycemia
HKDC1: hexokinase domain protein 1
LncRNA: Long noncoding RNA
ceRNA: competing endogenous RNA
HIF-1α: hypoxia-inducible factor-1α
RT: radiation therapy
EBRT: external beam radiation therapy
VBT: vaginal brachytherapy
HUVECs: human umbilical vein endothelial cells
AEs: adverse events
MDS: myelodysplastic syndrome
AML: acute myeloid leukemia
JNK: c-Jun N-terminal kinase
α-NETA: 2-(α-naphthoyl) ethyltrimethylammonium iodide
USC: Uterine-serous-carcinoma
HCC: hepatocellular carcinoma
ATC: anaplastic thyroid cancer
MMRd: MSI-H: mismatch repair deficient
p53abn: p53 abnormal type
p53wt: p53 wild type
HER2: human epidermal growth factor receptor 2

## References

[1] Sung H Ferlay J Siegel RL Laversanne M Soerjomataram I Jemal A. Global cancer statistics 2020: GLOBOCAN estimates of incidence and mortality worldwide for 36 cancers in 185 countries. CA Cancer J Clin (2021) 3(71):209–49. doi: 10.3322/caac.21660 33538338

[2] Gu B Shang X Yan M Li X Wang W Wang Q. Variations in incidence and mortality rates of endometrial cancer at the global, regional, and national levels, 1990-2019. Gynecol Oncol (2021) 161(2):537–80. doi: 10.1016/j.ygyno.2021.01.036 33551200

[3] Makker V MacKay H Ray-Coquard I Levine DA Westin SN Aoki D. Endometrial cancer. Nat Rev Dis Primers (2021) 1(7):88. doi: 10.1038/s41572-021-00324-8 PMC9421940 34887451

[4] McVicker L Cardwell CR Edge L McCluggage WG Quinn D Wylie J. Survival outcomes in endometrial cancer patients according to diabetes: A systematic review and meta-analysis. BMC Cancer (2022) 1(22):427. doi: 10.1186/s12885-022-09510-7 PMC9019948 35439978

[5] Clarke MA Long BJ Del MMA Arbyn M Bakkum-Gamez JN Wentzensen N. Association of endometrial cancer risk with postmenopausal bleeding in women: A systematic review and meta-analysis. JAMA Intern Med (2018) 9(178):1210–22. doi: 10.1001/jamainternmed.2018.2820 PMC6142981 30083701

[6] Crosbie EJ Kitson SJ McAlpine JN Mukhopadhyay A Powell ME Singh N. Endometrial cancer. Lancet (2022) 10333(399):1412–28. doi: 10.1016/S0140-6736(22)00323-3 35397864

[7] Oaknin A Bosse TJ Creutzberg CL Giornelli G Harter P Joly F. Endometrial cancer: ESMO Clinical Practice Guideline for diagnosis, treatment and follow-up. Ann Oncol (2022) 9(33):860–77. doi: 10.1016/j.annonc.2022.05.009 35690222

[8] Makker V Colombo N Casado HA Santin AD Colomba E Miller DS. Lenvatinib plus pembrolizumab for advanced endometrial cancer. N Engl J Med (2022) 5(386):437–48. doi: 10.1056/NEJMoa2108330 PMC11651366 35045221

[9] Heudel P Frenel JS Dalban C Bazan F Joly F Arnaud A. Safety and efficacy of the mTOR inhibitor, vistusertib, combined with anastrozole in patients with hormone Receptor-Positive recurrent or metastatic endometrial cancer: The VICTORIA multicenter, open-label, phase 1/2 randomized clinical trial. JAMA Oncol (2022) 7(8):1001–9. doi: 10.1001/jamaoncol.2022.1047 PMC9100474 35551299

[10] Lu KH Broaddus RR. Endometrial cancer. N Engl J Med (2020) 21(383):2053–64. doi: 10.1056/NEJMra1514010 33207095

[11] Siegel RL Miller KD Fuchs HE Jemal A. Cancer statistics, 2022. CA Cancer J Clin (2022) 1(72):7–33. doi: 10.3322/caac.21708 35020204

[12] Niu X Chen L Li Y Hu Z He F. Ferroptosis, necroptosis, and pyroptosis in the tumor microenvironment: Perspectives for immunotherapy of SCLC. Semin Cancer Biol (2022) Pt 3(86):273–85. doi: 10.1016/j.semcancer.2022.03.009 35288298

[13] Zindel J Kubes P. DAMPs, PAMPs, and LAMPs in immunity and sterile inflammation. Annu Rev Pathol (2020) 15):493–518. doi: 10.1146/annurev-pathmechdis-012419-032847 31675482

[14] Chen KW Monteleone M Boucher D Sollberger G Ramnath D Condon ND. Noncanonical inflammasome signaling elicits gasdermin D-dependent neutrophil extracellular traps. Sci Immunol (2018) 26(3):eaar6676. doi: 10.1126/sciimmunol.aar6676 30143554

[15] Lee MS Kwon H Lee EY Kim DJ Park JH Tesh VL. Shiga toxins activate the NLRP3 inflammasome pathway to promote both production of the proinflammatory cytokine interleukin-1β and apoptotic cell death. Infect Immun (2016) 1(84):172–86. doi: 10.1128/IAI.01095-15 PMC4694012 26502906

[16] Mariathasan S Weiss DS Newton K McBride J O'Rourke K Roose-Girma M. Cryopyrin activates the inflammasome in response to toxins and ATP. Nature (2006) 7081(440):228–32. doi: 10.1038/nature04515 16407890

[17] Kayagaki N Warming S Lamkanfi M Vande WL Louie S Dong J. Non-canonical inflammasome activation targets caspase-11. Nature (2011) 7371(479):117–21. doi: 10.1038/nature10558 22002608

[18] Kuriakose T Zheng M Neale G Kanneganti TD. IRF1 is a transcriptional regulator of ZBP1 promoting NLRP3 inflammasome activation and cell death during influenza virus infection. J Immunol (2018) 4(200):1489–95. doi: 10.4049/jimmunol.1701538 PMC6483084 29321274

[19] Doitsh G Galloway NL Geng X Yang Z Monroe KM Zepeda O. Cell death by pyroptosis drives CD4 T-cell depletion in HIV-1 infection. Nature (2014) 7484(505):509–14. doi: 10.1038/nature12940 PMC4047036 24356306

[20] Grotemeyer A Fischer JF Koprich JB Brotchie JM Blum R Volkmann J. Inflammasome inhibition protects dopaminergic neurons from α-synuclein pathology in a model of progressive Parkinson's disease. J Neuroinflamm (2023) 1(20):79. doi: 10.1186/s12974-023-02759-0 PMC10029189 36945016

[21] Coll RC Schroder K Pelegrín P. NLRP3 and pyroptosis blockers for treating inflammatory diseases. Trends Pharmacol Sci (2022) 8(43):653–68. doi: 10.1016/j.tips.2022.04.003 35513901

[22] Huang Y Jiang H Chen Y Wang X Yang Y Tao J. Tranilast directly targets NLRP3 to treat inflammasome-driven diseases. EMBO Mol Med (2018) 4(10). doi: 10.15252/emmm.201708689 PMC5887903 29531021

[23] van der Heijden T Kritikou E Venema W van Duijn J van Santbrink PJ Slütter B. NLRP3 inflammasome inhibition by MCC950 reduces atherosclerotic lesion development in apolipoprotein E-Deficient Mice-Brief report. Arterioscler Thromb Vasc Biol (2017) 8(37):1457–61. doi: 10.1161/ATVBAHA.117.309575 28596375

[24] Hu Y Wen Q Cai Y Liu Y Ma W Li Q. Alantolactone induces concurrent apoptosis and GSDME-dependent pyroptosis of anaplastic thyroid cancer through ROS mitochondria-dependent caspase pathway. Phytomedicine (2023) 108):154528. doi: 10.1016/j.phymed.2022.154528 36343549

[25] Shangguan F Zhou H Ma N Wu S Huang H Jin G. A novel mechanism of cannabidiol in suppressing hepatocellular carcinoma by inducing GSDME dependent pyroptosis. Front Cell Dev Biol (2021) 9):697832. doi: 10.3389/fcell.2021.697832 34350183 PMC8327166

[26] Yuan R Zhao W Wang QQ He J Han S Gao H. Cucurbitacin B inhibits non-small cell lung cancer in *vivo* and in *vitro* by triggering TLR4/NLRP3/GSDMD-dependent pyroptosis. Pharmacol Res (2021) 170):105748. doi: 10.1016/j.phrs.2021.105748 34217831

[27] Hou J Zhao R Xia W Chang CW You Y Hsu JM. PD-L1-mediated gasdermin C expression switches apoptosis to pyroptosis in cancer cells and facilitates tumour necrosis. Nat Cell Biol (2020) 10(22):1264–75. doi: 10.1038/s41556-020-0575-z PMC7653546 32929201

[28] Huo J Shen Y Zhang Y Shen L. BI 2536 induces gasdermin E-dependent pyroptosis in ovarian cancer. Front Oncol (2022) 12):963928. doi: 10.3389/fonc.2022.963928 36016611 PMC9396031

[29] Galluzzi L Vitale I Aaronson SA Abrams JM Adam D Agostinis P. Molecular mechanisms of cell death: Recommendations of the Nomenclature Committee on Cell Death 2018. Cell Death Differ (2018) 3(25):486–541. doi: 10.1038/s41418-017-0012-4 PMC5864239 29362479

[30] Lim M Jantaree P Naumann M. The conundrum of Helicobacter pylori-associated apoptosis in gastric cancer. Trends Cancer (2023) 9(8):679–90. doi: 10.1016/j.trecan.2023.04.012 37230895

[31] Yatim N Jusforgues-Saklani H Orozco S Schulz O Barreira DSR Reis ESC. RIPK1 and NF-κB signaling in dying cells determines cross-priming of CD8^+^ T cells. Science (2015) 6258(350):328–34. doi: 10.1126/science.aad0395 PMC4651449 26405229

[32] Yang WS Stockwell BR. Ferroptosis: Death by lipid peroxidation. Trends Cell Biol (2016) 3(26):165–76. doi: 10.1016/j.tcb.2015.10.014 PMC4764384 26653790

[33] Vargas J Hamasaki M Kawabata T Youle RJ Yoshimori T. The mechanisms and roles of selective autophagy in mammals. Nat Rev Mol Cell Biol (2023) 3(24):167–85. doi: 10.1038/s41580-022-00542-2 36302887

[34] Liu Z Wang C Yang J Zhou B Yang R Ramachandran R. Crystal structures of the Full-Length murine and human gasdermin d reveal mechanisms of autoinhibition, lipid binding, and oligomerization. Immunity (2019) 1(51):43–9. doi: 10.1016/j.immuni.2019.04.017 PMC6640092 31097341

[35] Ding J Wang K Liu W She Y Sun Q Shi J. Pore-forming activity and structural autoinhibition of the gasdermin family. Nature (2016) 535(7610):111–6. doi: 10.1038/nature18590 27281216

[36] Liu X Zhang Z Ruan J Pan Y Magupalli VG Wu H. Inflammasome-activated gasdermin D causes pyroptosis by forming membrane pores. Nature (2016) 7610(535):153–8. doi: 10.1038/nature18629 PMC5539988 27383986

[37] Xia S Zhang Z Magupalli VG Pablo JL Dong Y Vora SM. Gasdermin D pore structure reveals preferential release of mature interleukin-1. Nature (2021) 7860(593):607–11. doi: 10.1038/s41586-021-03478-3 PMC8588876 33883744

[38] Volchuk A Ye A Chi L Steinberg BE Goldenberg NM. Indirect regulation of HMGB1 release by gasdermin D. Nat Commun (2020) 1(11):4561. doi: 10.1038/s41467-020-18443-3 PMC7486936 32917873

[39] Broz P Dixit VM. Inflammasomes: Mechanism of assembly, regulation and signalling. Nat Rev Immunol (2016) 7(16):407–20. doi: 10.1038/nri.2016.58 27291964

[40] Chan AH Schroder K. Inflammasome signaling and regulation of interleukin-1 family cytokines. J Exp Med (2020) 1(217). doi: 10.1084/jem.20190314 PMC7037238 31611248

[41] Wei X Xie F Zhou X Wu Y Yan H Liu T. Role of pyroptosis in inflammation and cancer. Cell Mol Immunol (2022) 9(19):971–92. doi: 10.1038/s41423-022-00905-x PMC9376585 35970871

[42] Martinon F Burns K Tschopp J. The inflammasome: A molecular platform triggering activation of inflammatory caspases and processing of proIL-beta. Mol Cell (2002) 2(10):417–26. doi: 10.1016/s1097-2765(02)00599-3 12191486

[43] Próchnicki T Vasconcelos MB Robinson KS Mangan M De Graaf D Shkarina K. Mitochondrial damage activates the NLRP10 inflammasome. Nat Immunol (2023) 24(4):595–603. doi: 10.1038/s41590-023-01451-y 36941400

[44] Li D Wu M. Pattern recognition receptors in health and diseases. Signal Transduct Target Ther (2021) 1(6):291. doi: 10.1038/s41392-021-00687-0 PMC8333067 34344870

[45] Sharma BR Kanneganti TD. NLRP3 inflammasome in cancer and metabolic diseases. Nat Immunol (2021) 5(22):550–9. doi: 10.1038/s41590-021-00886-5 PMC8132572 33707781

[46] Bauernfeind FG Horvath G Stutz A Alnemri ES MacDonald K Speert D. Cutting edge: NF-kappaB activating pattern recognition and cytokine receptors license NLRP3 inflammasome activation by regulating NLRP3 expression. J Immunol (2009) 2(183):787–91. doi: 10.4049/jimmunol.0901363 PMC2824855 19570822

[47] Mathur A Feng S Hayward JA Ngo C Fox D Atmosukarto II. A multicomponent toxin from Bacillus cereus incites inflammation and shapes host outcome *via* the NLRP3 inflammasome. Nat Microbiol (2019) 2(4):362–74. doi: 10.1038/s41564-018-0318-0 PMC7685251 30531979

[48] Kuriakose T Man SM Malireddi RK Karki R Kesavardhana S Place DE. ZBP1/DAI is an innate sensor of influenza virus triggering the NLRP3 inflammasome and programmed cell death pathways. Sci Immunol (2016) 2(1):aag2045. doi: 10.1126/sciimmunol.aag2045 PMC5131924 27917412

[49] Karki R Man SM Malireddi R Gurung P Vogel P Lamkanfi M. Concerted activation of the AIM2 and NLRP3 inflammasomes orchestrates host protection against Aspergillus infection. Cell Host Microbe (2015) 3(17):357–68. doi: 10.1016/j.chom.2015.01.006 PMC4359672 25704009

[50] Lamkanfi M Malireddi RK Kanneganti TD. Fungal zymosan and mannan activate the cryopyrin inflammasome. J Biol Chem (2009) 31(284):20574–81. doi: 10.1074/jbc.M109.023689 PMC2742822 19509280

[51] Briard B Fontaine T Samir P Place DE Muszkieta L Malireddi R. Galactosaminogalactan activates the inflammasome to provide host protection. Nature (2020) 7839(588):688–92. doi: 10.1038/s41586-020-2996-z PMC8086055 33268895

[52] Swanson KV Deng M Ting JP. The NLRP3 inflammasome: Molecular activation and regulation to therapeutics. Nat Rev Immunol (2019) 8(19):477–89. doi: 10.1038/s41577-019-0165-0 PMC7807242 31036962

[53] Duewell P Kono H Rayner KJ Sirois CM Vladimer G Bauernfeind FG. NLRP3 inflammasomes are required for atherogenesis and activated by cholesterol crystals. Nature (2010) 7293(464):1357–61. doi: 10.1038/nature08938 PMC2946640 20428172

[54] Muñoz-Planillo R Kuffa P Martínez-Colón G Smith BL Rajendiran TM Núñez G. K^+^ efflux is the common trigger of NLRP3 inflammasome activation by bacterial toxins and particulate matter. Immunity (2013) 6(38):1142–53. doi: 10.1016/j.immuni.2013.05.016 PMC3730833 23809161

[55] He Y Zeng MY Yang D Motro B Núñez G. NEK7 is an essential mediator of NLRP3 activation downstream of potassium efflux. Nature (2016) 7590(530):354–7. doi: 10.1038/nature16959 PMC4810788 26814970

[56] Triantafilou K Hughes TR Triantafilou M Morgan BP. The complement membrane attack complex triggers intracellular Ca2+ fluxes leading to NLRP3 inflammasome activation. J Cell Sci (2013) Pt 13(126):2903–13. doi: 10.1242/jcs.124388 23613465

[57] Hornung V Bauernfeind F Halle A Samstad EO Kono H Rock KL. Silica crystals and aluminum salts activate the NALP3 inflammasome through phagosomal destabilization. Nat Immunol (2008) 8(9):847–56. doi: 10.1038/ni.1631 PMC2834784 18604214

[58] Wen H Gris D Lei Y Jha S Zhang L Huang MT. Fatty acid-induced NLRP3-ASC inflammasome activation interferes with insulin signaling. Nat Immunol (2011) 5(12):408–15. doi: 10.1038/ni.2022 PMC4090391 21478880

[59] Bronner DN Abuaita BH Chen X Fitzgerald KA Nuñez G He Y. Endoplasmic reticulum stress activates the inflammasome *via* NLRP3- and Caspase-2-Driven mitochondrial damage. Immunity (2015) 3(43):451–62. doi: 10.1016/j.immuni.2015.08.008 PMC4582788 26341399

[60] Zhou Y Tong Z Jiang S Zheng W Zhao J Zhou X. The roles of endoplasmic reticulum in NLRP3 inflammasome activation. Cells (2020) 5(9). doi: 10.3390/cells9051219 PMC7291288 32423023

[61] Andreeva L David L Rawson S Shen C Pasricha T Pelegrin P. NLRP3 cages revealed by full-length mouse NLRP3 structure control pathway activation. Cell (2021) 26(184):6299–312. doi: 10.1016/j.cell.2021.11.011 PMC8763037 34861190

[62] Chen J Chen ZJ. PtdIns4P on dispersed trans-Golgi network mediates NLRP3 inflammasome activation. Nature (2018) 7734(564):71–6. doi: 10.1038/s41586-018-0761-3 PMC9402428 30487600

[63] Zhang Z Venditti R Ran L Liu Z Vivot K Schürmann A. Distinct changes in endosomal composition promote NLRP3 inflammasome activation. Nat Immunol (2023) 1(24):30–41. doi: 10.1038/s41590-022-01355-3 PMC9810532 36443515

[64] Sharif H Wang L Wang WL Magupalli VG Andreeva L Qiao Q. Structural mechanism for NEK7-licensed activation of NLRP3 inflammasome. Nature (2019) 7761(570):338–43. doi: 10.1038/s41586-019-1295-z PMC6774351 31189953

[65] Xiao L Magupalli VG Wu H. Cryo-EM structures of the active NLRP3 inflammasome disc. Nature (2023) 7944(613):595–600. doi: 10.1038/s41586-022-05570-8 PMC10091861 36442502

[66] Bauernfried S Scherr MJ Pichlmair A Duderstadt KE Hornung V. Human NLRP1 is a sensor for double-stranded RNA. Science (2021) 6528(371):eabd0811. doi: 10.1126/science.abd0811 33243852

[67] Robinson KS Teo D Tan KS Toh GA Ong HH Lim CK. Enteroviral 3C protease activates the human NLRP1 inflammasome in airway epithelia. Science (2020) 6521(370):eaay2002. doi: 10.1126/science.aay2002 33093214

[68] Okondo MC Rao SD Taabazuing CY Chui AJ Poplawski SE Johnson DC. Inhibition of dpp8/9 activates the nlrp1b inflammasome. Cell Chem Biol (2018) 3(25):262–7. doi: 10.1016/j.chembiol.2017.12.013 PMC5856610 29396289

[69] Cirelli KM Gorfu G Hassan MA Printz M Crown D Leppla SH. Inflammasome sensor NLRP1 controls rat macrophage susceptibility to Toxoplasma gondii. PloS Pathog (2014) 3(10):e1003927. doi: 10.1371/journal.ppat.1003927 PMC3953412 24626226

[70] de Vasconcelos NM Vliegen G Gonçalves A De Hert E Martín-Pérez R Van Opdenbosch N. DPP8/DPP9 inhibition elicits canonical Nlrp1b inflammasome hallmarks in murine macrophages. Life Sci Alliance (2019) 1(2). doi: 10.26508/lsa.201900313 PMC6362307 30718379

[71] Sandstrom A Mitchell PS Goers L Mu EW Lesser CF Vance RE. Functional degradation: A mechanism of NLRP1 inflammasome activation by diverse pathogen enzymes. Science (2019) 6435(364). doi: 10.1126/science.aau1330 PMC6532986 30872533

[72] D'Osualdo A Weichenberger CX Wagner RN Godzik A Wooley J Reed JC. CARD8 and NLRP1 undergo autoproteolytic processing through a ZU5-like domain. PloS One (2011) 11(6):e27396. doi: 10.1371/journal.pone.0027396 PMC3210808 22087307

[73] Chui AJ Okondo MC Rao SD Gai K Griswold AR Johnson DC. N-terminal degradation activates the NLRP1B inflammasome. Science (2019) 6435(364):82–5. doi: 10.1126/science.aau1208 PMC6610862 30872531

[74] Huang M Zhang X Toh GA Gong Q Wang J Han Z. Structural and biochemical mechanisms of NLRP1 inhibition by DPP9. Nature (2021) 7856(592):773–7. doi: 10.1038/s41586-021-03320-w PMC8081665 33731929

[75] Robert HL David L Li Y Griswold AR Ruan J Sharif H. Mechanism of filament formation in UPA-promoted CARD8 and NLRP1 inflammasomes. Nat Commun (2021) 1(12):189. doi: 10.1038/s41467-020-20320-y PMC7794386 33420033

[76] Gong Q Robinson K Xu C Huynh PT Chong K Tan E. Structural basis for distinct inflammasome complex assembly by human NLRP1 and CARD8. Nat Commun (2021) 1(12):188. doi: 10.1038/s41467-020-20319-5 PMC7794362 33420028

[77] Kofoed EM Vance RE. Innate immune recognition of bacterial ligands by NAIPs determines inflammasome specificity. Nature (2011) 7366(477):592–5. doi: 10.1038/nature10394 PMC3184209 21874021

[78] Hu Z Zhou Q Zhang C Fan S Cheng W Zhao Y. Structural and biochemical basis for induced self-propagation of NLRC4. Science (2015) 6259(350):399–404. doi: 10.1126/science.aac5489 26449475

[79] Karki R Lee E Place D Samir P Mavuluri J Sharma BR. IRF8 regulates transcription of naips for NLRC4 inflammasome activation. Cell (2018) 4(173):920–33. doi: 10.1016/j.cell.2018.02.055 PMC5935577 29576451

[80] Liu W Liu X Li Y Zhao J Liu Z Hu Z. LRRK2 promotes the activation of NLRC4 inflammasome during Salmonella Typhimurium infection. J Exp Med (2017) 10(214):3051–66. doi: 10.1084/jem.20170014 PMC5626397 28821568

[81] Qu Y Misaghi S Izrael-Tomasevic A Newton K Gilmour LL Lamkanfi M. Phosphorylation of NLRC4 is critical for inflammasome activation. Nature (2012) 7421(490):539–42. doi: 10.1038/nature11429 22885697

[82] Jin T Perry A Jiang J Smith P Curry JA Unterholzner L. Structures of the HIN domain:DNA complexes reveal ligand binding and activation mechanisms of the AIM2 inflammasome and IFI16 receptor. Immunity (2012) 4(36):561–71. doi: 10.1016/j.immuni.2012.02.014 PMC3334467 22483801

[83] Zhu Q Man SM Karki R Malireddi R Kanneganti TD. Detrimental Type I Interferon Signaling Dominates Protective AIM2 Inflammasome Responses during Francisella novicida Infection. Cell Rep (2018) 12(22):3168–74. doi: 10.1016/j.celrep.2018.02.096 PMC6204211 29562174

[84] Man SM Karki R Malireddi RK Neale G Vogel P Yamamoto M. The transcription factor IRF1 and guanylate-binding proteins target activation of the AIM2 inflammasome by Francisella infection. Nat Immunol (2015) 5(16):467–75. doi: 10.1038/ni.3118 PMC4406811 25774715

[85] Hu B Jin C Li HB Tong J Ouyang X Cetinbas NM. The DNA-sensing AIM2 inflammasome controls radiation-induced cell death and tissue injury. Science (2016) 6313(354):765–8. doi: 10.1126/science.aaf7532 PMC5640175 27846608

[86] Dang EV McDonald JG Russell DW Cyster JG. Oxysterol restraint of cholesterol synthesis prevents AIM2 inflammasome activation. Cell (2017) 5(171):1057–71. doi: 10.1016/j.cell.2017.09.029 PMC5693620 29033131

[87] Chung LK Park YH Zheng Y Brodsky IE Hearing P Kastner DL. The yersinia virulence factor YopM hijacks host kinases to inhibit type III Effector-Triggered activation of the pyrin inflammasome. Cell Host Microbe (2016) 3(20):296–306. doi: 10.1016/j.chom.2016.07.018 PMC5025386 27569559

[88] Xu H Yang J Gao W Li L Li P Zhang L. Innate immune sensing of bacterial modifications of Rho GTPases by the Pyrin inflammasome. Nature (2014) 7517(513):237–41. doi: 10.1038/nature13449 24919149

[89] Park YH Wood G Kastner DL Chae JJ. Pyrin inflammasome activation and RhoA signaling in the autoinflammatory diseases FMF and HIDS. Nat Immunol (2016) 8(17):914–21. doi: 10.1038/ni.3457 PMC4955684 27270401

[90] Viganò E Diamond CE Spreafico R Balachander A Sobota RM Mortellaro A. Human caspase-4 and caspase-5 regulate the one-step non-canonical inflammasome activation in monocytes. Nat Commun (2015) 6):8761. doi: 10.1038/ncomms9761 26508369 PMC4640152

[91] Kayagaki N Stowe IB Lee BL O'Rourke K Anderson K Warming S. Caspase-11 cleaves gasdermin D for non-canonical inflammasome signalling. Nature (2015) 7575(526):666–71. doi: 10.1038/nature15541 26375259

[92] Pfalzgraff A Weindl G. Intracellular lipopolysaccharide sensing as a potential therapeutic target for sepsis. Trends Pharmacol Sci (2019) 3(40):187–97. doi: 10.1016/j.tips.2019.01.001 30691865

[93] Broz P Pelegrín P Shao F. The gasdermins, a protein family executing cell death and inflammation. Nat Rev Immunol (2020) 3(20):143–57. doi: 10.1038/s41577-019-0228-2 31690840

[94] Yang D He Y Muñoz-Planillo R Liu Q Núñez G. Caspase-11 requires the pannexin-1 channel and the purinergic P2X7 pore to mediate pyroptosis and endotoxic shock. Immunity (2015) 5(43):923–32. doi: 10.1016/j.immuni.2015.10.009 PMC4795157 26572062

[95] Moretti J Jia B Hutchins Z Roy S Yip H Wu J. Caspase-11 interaction with NLRP3 potentiates the noncanonical activation of the NLRP3 inflammasome. Nat Immunol (2022) 5(23):705–17. doi: 10.1038/s41590-022-01192-4 PMC9106893 35487985

[96] Zhu F Ma J Li W Liu Q Qin X Qian Y. The orphan receptor Nur77 binds cytoplasmic LPS to activate the non-canonical NLRP3 inflammasome. Immunity (2023) 4(56):753–67. doi: 10.1016/j.immuni.2023.03.003 37001519

[97] David L Taieb F Pénary M Bordignon PJ Planès R Bagayoko S. Outer membrane vesicles produced by pathogenic strains of Escherichia coli block autophagic flux and exacerbate inflammasome activation. Autophagy (2022) 12(18):2913–25. doi: 10.1080/15548627.2022.2054040 PMC9673956 35311462

[98] Rathinam VA Vanaja SK Waggoner L Sokolovska A Becker C Stuart LM. TRIF licenses caspase-11-dependent NLRP3 inflammasome activation by gram-negative bacteria. Cell (2012) 3(150):606–19. doi: 10.1016/j.cell.2012.07.007 PMC3660860 22819539

[99] Wandel MP Kim BH Park ES Boyle KB Nayak K Lagrange B. Guanylate-binding proteins convert cytosolic bacteria into caspase-4 signaling platforms. Nat Immunol (2020) 8(21):880–91. doi: 10.1038/s41590-020-0697-2 PMC7381384 32541830

[100] Chu LH Indramohan M Ratsimandresy RA Gangopadhyay A Morris EP Monack DM. The oxidized phospholipid oxPAPC protects from septic shock by targeting the non-canonical inflammasome in macrophages. Nat Commun (2018) 1(9):996. doi: 10.1038/s41467-018-03409-3 PMC5843631 29520027

[101] Zanoni I Tan Y Di Gioia M Broggi A Ruan J Shi J. An endogenous caspase-11 ligand elicits interleukin-1 release from living dendritic cells. Science (2016) 6290(352):1232–6. doi: 10.1126/science.aaf3036 PMC5111085 27103670

[102] Li Z Liu W Fu J Cheng S Xu Y Wang Z. Shigella evades pyroptosis by arginine ADP-riboxanation of caspase-11. Nature (2021) 7884(599):290–5. doi: 10.1038/s41586-021-04020-1 34671164

[103] Kayagaki N Lee BL Stowe IB Kornfeld OS O'Rourke K Mirrashidi KM. IRF2 transcriptionally induces GSDMD expression for pyroptosis. Sci Signal (2019) 582(12):eaax4917. doi: 10.1126/scisignal.aax4917 31113851

[104] Deng W Bai Y Deng F Pan Y Mei S Zheng Z. Streptococcal pyrogenic exotoxin B cleaves GSDMA and triggers pyroptosis. Nature (2022) 7897(602):496–502. doi: 10.1038/s41586-021-04384-4 PMC9703647 35110732

[105] Zhou Z He H Wang K Shi X Wang Y Su Y. Granzyme a from cytotoxic lymphocytes cleaves GSDMB to trigger pyroptosis in target cells. Science (2020) 6494(368):eaaz7548. doi: 10.1126/science.aaz7548 32299851

[106] Lu YC Tsai YH Chan YH Hu CJ Huang CY Xiao R. Gene therapy with a synthetic adeno-associated viral vector improves audiovestibular phenotypes in Pjvk-mutant mice. JCI Insight (2022) 20(7). doi: 10.1172/jci.insight.152941 PMC9714786 36278489

[107] LaRock DL Johnson AF Wilde S Sands JS Monteiro MP LaRock CN. Group a Streptococcus induces GSDMA-dependent pyroptosis in keratinocytes. Nature (2022) 7910(605):527–31. doi: 10.1038/s41586-022-04717-x PMC9186297 35545676

[108] Chen Q Shi P Wang Y Zou D Wu X Wang D. GSDMB promotes non-canonical pyroptosis by enhancing caspase-4 activity. J Mol Cell Biol (2019) 6(11):496–508. doi: 10.1093/jmcb/mjy056 PMC6734491 30321352

[109] Hansen JM de Jong MF Wu Q Zhang LS Heisler DB Alto LT. Pathogenic ubiquitination of GSDMB inhibits NK cell bactericidal functions. Cell (2021) 12(184):3178–91. doi: 10.1016/j.cell.2021.04.036 PMC8221529 34022140

[110] Zhong X Zeng H Zhou Z Su Y Cheng H Hou Y. Structural mechanisms for regulation of GSDMB pore-forming activity. Nature (2023) 616(7957):598–605. doi: 10.1038/s41586-023-05872-5 36991125

[111] Hsu SK Li CY Lin IL Syue WJ Chen YF Cheng KC. Inflammation-related pyroptosis, a novel programmed cell death pathway, and its crosstalk with immune therapy in cancer treatment. Theranostics (2021) 18(11):8813–35. doi: 10.7150/thno.62521 PMC8419056 34522213

[112] Taabazuing CY Okondo MC Bachovchin DA. Pyroptosis and apoptosis pathways engage in bidirectional crosstalk in monocytes and macrophages. Cell Chem Biol (2017) 4(24):507–14. doi: 10.1016/j.chembiol.2017.03.009 PMC5467448 28392147

[113] Rogers C Erkes DA Nardone A Aplin AE Fernandes-Alnemri T Alnemri ES. Gasdermin pores permeabilize mitochondria to augment caspase-3 activation during apoptosis and inflammasome activation. Nat Commun (2019) 1(10):1689. doi: 10.1038/s41467-019-09397-2 PMC6459836 30976076

[114] Orning P Weng D Starheim K Ratner D Best Z Lee B. Pathogen blockade of TAK1 triggers caspase-8-dependent cleavage of gasdermin D and cell death. Science (2018) 362(6418):1064–9. doi: 10.1126/science.aau2818 PMC6522129 30361383

[115] Sarhan J Liu BC Muendlein HI Li P Nilson R Tang AY. Caspase-8 induces cleavage of gasdermin D to elicit pyroptosis during Yersinia infection. Proc Natl Acad Sci U.S.A. (2018) 46(115):E10888–97. doi: 10.1073/pnas.1809548115 PMC6243247 30381458

[116] Zheng Z Deng W Bai Y Miao R Mei S Zhang Z. The lysosomal Rag-Ragulator complex licenses RIPK1 and caspase-8-mediated pyroptosis by yersinia. Science (2021) 6549(372):eabg0269. doi: 10.1126/science.abg0269 PMC8769499 35058659

[117] Wang Y Gao W Shi X Ding J Liu W He H. Chemotherapy drugs induce pyroptosis through caspase-3 cleavage of a gasdermin. Nature (2017) 7661(547):99–103. doi: 10.1038/nature22393 28459430

[118] Rogers C Fernandes-Alnemri T Mayes L Alnemri D Cingolani G Alnemri ES. Cleavage of DFNA5 by caspase-3 during apoptosis mediates progression to secondary necrotic/pyroptotic cell death. Nat Commun (2017) 8):14128. doi: 10.1038/ncomms14128 28045099 PMC5216131

[119] Liu Y Fang Y Chen X Wang Z Liang X Zhang T. Gasdermin E-mediated target cell pyroptosis by CAR T cells triggers cytokine release syndrome. Sci Immunol (2020) 43(5):eaax7969. doi: 10.1126/sciimmunol.aax7969 31953257

[120] Lin J Sun S Zhao K Gao F Wang R Li Q. Oncolytic Parapoxvirus induces Gasdermin E-mediated pyroptosis and activates antitumor immunity. Nat Commun (2023) 1(14):224. doi: 10.1038/s41467-023-35917-2 PMC9840172 36641456

[121] Li Y Yao N Gao Y Wang Y Bai L Xu J. MiR-1224-5p attenuates polycystic ovary syndrome through inhibiting NOD-like receptor protein 3 inflammasome activation *via* targeting Forkhead box O 1. Bioengineered (2021) 1(12):8555–69. doi: 10.1080/21655979.2021.1987125 PMC8806973 34637688

[122] Yu S Zhao N He M Zhang K Bi X. MiRNA-214 promotes the pyroptosis and inhibits the proliferation of cervical cancer cells *via* regulating the expression of NLRP3. Cell Mol Biol (Noisy-le-grand) (2020) 6(66):59–64. doi: 10.14715/cmb/2020.66.6.11 33040786

[123] Fang X Wang Y Zhang Y Li Y Kwak-Kim J Wu L. NLRP3 inflammasome and its critical role in gynecological disorders and obstetrical complications. Front Immunol (2020) 11):555826. doi: 10.3389/fimmu.2020.555826 33584639 PMC7876052

[124] Hang Y Tan L Chen Q Liu Q Jin Y. E3 ubiquitin ligase TRIM24 deficiency promotes NLRP3/caspase-1/IL-1β-mediated pyroptosis in endometriosis. Cell Biol Int (2021) 7(45):1561–70. doi: 10.1002/cbin.11592 33724611

[125] Guo J Ye F Xie W Zhang X Zeng R Sheng W. The HOXC-AS2/miR-876-5p/HKDC1 axis regulates endometrial cancer progression in a high glucose-related tumor microenvironment. Cancer Sci (2022) 7(113):2297–310. doi: 10.1111/cas.15384 PMC9277262 35485648

[126] Yang Y Liu PY Bao W Chen SJ Wu FS Zhu PY. Hydrogen inhibits endometrial cancer growth *via* a ROS/NLRP3/caspase-1/GSDMD-mediated pyroptotic pathway. BMC Cancer (2020) 1(20):28. doi: 10.1186/s12885-019-6491-6 PMC6954594 31924176

[127] Murakami M Osuka S Muraoka A Hayashi S Bayasula Kasahara Y. Effectiveness of NLRP3 Inhibitor as a Non-Hormonal Treatment for ovarian endometriosis. Reprod Biol Endocrinol (2022) 1(20):58. doi: 10.1186/s12958-022-00924-3 PMC8966161 35351143

[128] Amant F Moerman P Neven P Timmerman D Van Limbergen E Vergote I. Endometrial cancer... Lancet (2005) 9484(366):491–505. doi: 10.1016/S0140-6736(05)67063-8 16084259

[129] De Nola R Menga A Castegna A Loizzi V Ranieri G Cicinelli E. The crowded crosstalk between cancer cells and stromal microenvironment in gynecological Malignancies: Biological pathways and therapeutic implication. Int J Mol Sci (2019) 10(20). doi: 10.3390/ijms20102401 PMC6567055 31096567

[130] Hapangama DK Kamal AM Bulmer JN. Estrogen receptor β: The guardian of the endometrium. Hum Reprod Update (2015) 2(21):174–93. doi: 10.1093/humupd/dmu053 25305176

[131] Matthews J Wihlén B Tujague M Wan J Ström A Gustafsson JA. Estrogen receptor (ER) beta modulates ERalpha-mediated transcriptional activation by altering the recruitment of c-Fos and c-Jun to estrogen-responsive promoters. Mol Endocrinol (2006) 3(20):534–43. doi: 10.1210/me.2005-0140 16293641

[132] Liu SG Wu XX Hua T Xin XY Feng DL Chi SQ. NLRP3 inflammasome activation by estrogen promotes the progression of human endometrial cancer. Onco Targets Ther (2019) 12):6927–36. doi: 10.2147/OTT.S218240 PMC6717726 31695408

[133] Hu J Pei W Jiang M Huang Y Dong F Jiang Z. DFNA5 regulates immune cells infiltration and exhaustion. Cancer Cell Int (2022) 1(22):107. doi: 10.1186/s12935-022-02487-0 PMC8897971 35248047

[134] Croes L Beyens M Fransen E Ibrahim J Vanden BW Suls A. Large-scale analysis of DFNA5 methylation reveals its potential as biomarker for breast cancer. Clin Epigenet (2018) 10):51. doi: 10.1186/s13148-018-0479-y PMC5896072 29682089

[135] Xia X Wang X Cheng Z Qin W Lei L Jiang J. The role of pyroptosis in cancer: Pro-cancer or pro-"host"? Cell Death Dis (2019) 9(10):650. doi: 10.1038/s41419-019-1883-8 PMC6733901 31501419

[136] Chen Y Liao Y Du Q Shang C Qin S Lee K. Roles of Pyroptosis-Related gene signature in prediction of endometrial cancer outcomes. Front Med (Lausanne) (2022) 9):822806. doi: 10.3389/fmed.2022.822806 35299842 PMC8920994

[137] Zhang Z Zhang Y Xia S Kong Q Li S Liu X. Gasdermin E suppresses tumour growth by activating anti-tumour immunity. Nature (2020) 7799(579):415–20. doi: 10.1038/s41586-020-2071-9 PMC7123794 32188940

[138] Sahai E Astsaturov I Cukierman E DeNardo DG Egeblad M Evans RM. A framework for advancing our understanding of cancer-associated fibroblasts. Nat Rev Cancer (2020) 3(20):174–86. doi: 10.1038/s41568-019-0238-1 PMC7046529 31980749

[139] Shikata K Ninomiya T Kiyohara Y. Diabetes mellitus and cancer risk: Review of the epidemiological evidence. Cancer Sci (2013) 1(104):9–14. doi: 10.1111/cas.12043 PMC7657146 23066889

[140] Saed L Varse F Baradaran HR Moradi Y Khateri S Friberg E. The effect of diabetes on the risk of endometrial Cancer: An updated a systematic review and meta-analysis. BMC Cancer (2019) 1(19):527. doi: 10.1186/s12885-019-5748-4 PMC6544993 31151429

[141] Esposito K Chiodini P Capuano A Bellastella G Maiorino MI Giugliano D. Metabolic syndrome and endometrial cancer: A meta-analysis. Endocrine (2014) 1(45):28–36. doi: 10.1007/s12020-013-9973-3 23640372

[142] Rosato V Zucchetto A Bosetti C Dal Maso L Montella M Pelucchi C. Metabolic syndrome and endometrial cancer risk. Ann Oncol (2011) 4(22):884–9. doi: 10.1093/annonc/mdq464 20937645

[143] Moukarzel LA Ferrando L Stylianou A Lobaugh S Wu M Nobre SP. Impact of obesity and white adipose tissue inflammation on the omental microenvironment in endometrial cancer. Cancer-Am. Cancer Soc (2022) 18(128):3297–309. doi: 10.1002/cncr.34356 PMC9976596 35793549

[144] Renehan AG Zwahlen M Egger M. Adiposity and cancer risk: New mechanistic insights from epidemiology. Nat Rev Cancer (2015) 8(15):484–98. doi: 10.1038/nrc3967 26205341

[145] Zhou F Zhao F Huang Q Lin X Zhang S Dai Y. NLRP3 activated macrophages promote endometrial stromal cells migration in endometriosis. J Reprod Immunol (2022) 152):103649. doi: 10.1016/j.jri.2022.103649 35714422

[146] Ohsawa I Ishikawa M Takahashi K Watanabe M Nishimaki K Yamagata K. Hydrogen acts as a therapeutic antioxidant by selectively reducing cytotoxic oxygen radicals. Nat Med (2007) 6(13):688–94. doi: 10.1038/nm1577 17486089

[147] Tao G Liu F Jin Z Liu B Wang H Li D. A strategy of local hydrogen capture and catalytic hydrogenation for enhanced therapy of chronic liver diseases. Theranostics (2023) 8(13):2455–70. doi: 10.7150/thno.80494 PMC10196827 37215568

[148] Li K Yin H Duan Y Lai P Cai Y Wei Y. Pre-inhalation of hydrogen-rich gases protect against caerulein-induced mouse acute pancreatitis while enhance the pancreatic Hsp60 protein expression. BMC Gastroenterol (2021) 1(21):178. doi: 10.1186/s12876-021-01640-9 PMC8056676 33874887

[149] Wang ST Bao C He Y Tian X Yang Y Zhang T. Hydrogen gas (XEN) inhalation ameliorates airway inflammation in asthma and COPD patients. QJM (2020) 12(113):870–5. doi: 10.1093/qjmed/hcaa164 PMC7785302 32407476

[150] Zhu P Shen L Ren Q Zeng Q He X. Prognostic and clinicopathological significance of Hypoxia-Inducible factor-1α in endometrial cancer: A Meta-Analysis. Front Oncol (2020) 10):587420. doi: 10.3389/fonc.2020.587420 33304847 PMC7693720

[151] An WG Kanekal M Simon MC Maltepe E Blagosklonny MV Neckers LM. Stabilization of wild-type p53 by hypoxia-inducible factor 1alpha. Nature (1998) 6674(392):405–8. doi: 10.1038/32925 9537326

[152] Horrée N Gort EH van der Groep P Heintz AP Vooijs M van Diest PJ. Hypoxia-inducible factor 1 alpha is essential for hypoxic p27 induction in endometrioid endometrial carcinoma. J Pathol (2008) 1(214):38–45. doi: 10.1002/path.2244 17985331

[153] Su P Yu L Mao X Sun P. Role of HIF-1α/ERRα in enhancing cancer cell metabolism and promoting resistance of endometrial cancer cells to pyroptosis. Front Oncol (2022) 12:881252. doi: 10.3389/fonc.2022.881252 35800058 PMC9253301

[154] Creutzberg CL van Putten WL Koper PC Lybeert ML Jobsen JJ Wárlám-Rodenhuis CC. Surgery and postoperative radiotherapy versus surgery alone for patients with stage-1 endometrial carcinoma: Multicentre randomised trial. PORTEC Study Group. Post Operative Radiation Therapy in Endometrial Carcinoma. Lancet (2000) 9213(355):1404–11. doi: 10.1016/s0140-6736(00)02139-5 10791524

[155] Keys HM Roberts JA Brunetto VL Zaino RJ Spirtos NM Bloss JD. A phase III trial of surgery with or without adjunctive external pelvic radiation therapy in intermediate risk endometrial adenocarcinoma: A Gynecologic Oncology Group study. Gynecol. Oncol (2004) 3(92):744–51. doi: 10.1016/j.ygyno.2003.11.048 14984936

[156] Li C Tian M Gou Q Jia YR Su X. Connexin43 modulates X-Ray-Induced pyroptosis in human umbilical vein endothelial cells. Biomed Environ Sci (2019) 3(32):177–88. doi: 10.3967/bes2019.025 30987692

[157] Pelegrin P Surprenant A. Pannexin-1 mediates large pore formation and interleukin-1beta release by the ATP-gated P2X7 receptor. EMBO J (2006) 21(25):5071–82. doi: 10.1038/sj.emboj.7601378 PMC1630421 17036048

[158] Wu D Han R Deng S Liu T Zhang T Xie H. Protective effects of flagellin a N/C against Radiation-Induced NLR pyrin domain containing 3 Inflammasome-Dependent pyroptosis in intestinal cells. Int J Radiat Oncol Biol Phys (2018) 1(101):107–17. doi: 10.1016/j.ijrobp.2018.01.035 29456024

[159] Zhang Z Zhang H Li D Zhou X Qin Q Zhang Q. Caspase-3-mediated GSDME induced Pyroptosis in breast cancer cells through the ROS/JNK signalling pathway. J Cell Mol Med (2021) 17(25):8159–68. doi: 10.1111/jcmm.16574 PMC8419174 34369076

[160] Miller DS Filiaci VL Mannel RS Cohn DE Matsumoto T Tewari KS. Carboplatin and paclitaxel for advanced endometrial cancer: Final overall survival and adverse event analysis of a phase III trial (NRG Oncology/GOG0209). J Clin Oncol (2020) 33(38):3841–50. doi: 10.1200/JCO.20.01076 PMC7676887 33078978

[161] Nagao S Nishio S Michimae H Tanabe H Okada S Otsuki T. Applicability of the concept of "platinum sensitivity" to recurrent endometrial cancer: The SGSG-012/GOTIC-004/Intergroup study. Gynecol. Oncol (2013) 3(131):567–73. doi: 10.1016/j.ygyno.2013.09.021 24076450

[162] Chen D Guo S Tang X Rong Y Bo H Shen H. Combination of ruthenium (II) polypyridyl complex Δ-Ru1 and Taxol enhances the anti-cancer effect on Taxol-resistant cancer cells through Caspase-1/GSDMD-mediated pyroptosis. J Inorg Biochem (2022) 230):111749. doi: 10.1016/j.jinorgbio.2022.111749 35144218

[163] Ball B Zeidan A Gore SD Prebet T. Hypomethylating agent combination strategies in myelodysplastic syndromes: Hopes and shortcomings. Leuk Lymphoma (2017) 5(58):1022–36. doi: 10.1080/10428194.2016.1228927 PMC5785935 27654579

[164] Ma J Ge Z. Comparison between decitabine and azacitidine for patients with acute myeloid leukemia and Higher-Risk myelodysplastic syndrome: A systematic review and network Meta-Analysis. Front Pharmacol (2021) 12):701690. doi: 10.3389/fphar.2021.701690 34483903 PMC8416074

[165] Zhang CC Li CG Wang YF Xu LH He XH Zeng QZ. Chemotherapeutic paclitaxel and cisplatin differentially induce pyroptosis in A549 lung cancer cells via caspase-3/GSDME activation. Apoptosis (2019) 3-4(24):312–25. doi: 10.1007/s10495-019-01515-1 30710195

[166] Yu J Li S Qi J Chen Z Wu Y Guo J. Cleavage of GSDME by caspase-3 determines lobaplatin-induced pyroptosis in colon cancer cells. Cell Death Dis (2019) 3(10):193. doi: 10.1038/s41419-019-1441-4 PMC6389936 30804337

[167] Qiao L Wu X Zhang J Liu L Sui X Zhang R. A-NETA induces pyroptosis of epithelial ovarian cancer cells through the GSDMD/caspase-4 pathway. FASEB J (2019) 11(33):12760–7. doi: 10.1096/fj.201900483RR 31480859

[168] Tong W Guo J Yang C. Tanshinone II a enhances pyroptosis and represses cell proliferation of HeLa cells by regulating miR-145/GSDMD signaling pathway. Biosci Rep (2020) 4(40). doi: 10.1042/BSR20200259 PMC7160242 32232409

[169] Ding Q Zhang W Cheng C Mo F Chen L Peng G. Dioscin inhibits the growth of human osteosarcoma by inducing G2/M-phase arrest, apoptosis, and GSDME-dependent cell death in *vitro* and in *vivo* . J Cell Physiol (2020) 3(235):2911–24. doi: 10.1002/jcp.29197 31535374

[170] Jiang M Wu Y Qi L Li L Song D Gan J. Dihydroartemisinin mediating PKM2-caspase-8/3-GSDME axis for pyroptosis in esophageal squamous cell carcinoma. Chem Biol Interact (2021) 350):109704. doi: 10.1016/j.cbi.2021.109704 34655567

[171] Hu Z Lai Y Ma C Zuo L Xiao G Gao H. Cordyceps militaris extract induces apoptosis and pyroptosis *via* caspase-3/PARP/GSDME pathways in A549 cell line. Food Sci Nutr (2022) 1(10):21–38. doi: 10.1002/fsn3.2636 PMC8751435 35035907

[172] Chu Q Jiang Y Zhang W Xu C Du W Tuguzbaeva G. Pyroptosis is involved in the pathogenesis of human hepatocellular carcinoma. Oncotarget (2016) 51(7):84658–65. doi: 10.18632/oncotarget.12384 PMC5356689 27705930

[173] Zhang Y Yang J Wen Z Chen X Yu J Yuan D. A novel 3',5'-diprenylated chalcone induces concurrent apoptosis and GSDME-dependent pyroptosis through activating PKCδ/JNK signal in prostate cancer. Aging (Albany NY) (2020) 10(12):9103–24. doi: 10.18632/aging.103178 PMC7288973 32427575

[174] Chen L Li Q Zheng Z Xie J Lin X Jiang C. Design and optimize N-substituted EF24 as effective and low toxicity NF-κB inhibitor for lung cancer therapy *via* apoptosis-to-pyroptosis switch. Chem Biol Drug Des (2019) 1(94):1368–77. doi: 10.1111/cbdd.13514 30873716

[175] Fader AN Roque DM Siegel E Buza N Hui P Abdelghany O. Randomized phase II trial of Carboplatin-Paclitaxel compared with Carboplatin-Paclitaxel-Trastuzumab in advanced (Stage III-IV) or recurrent uterine serous carcinomas that overexpress Her2/Neu (NCT01367002): Updated overall survival analysis. Clin Cancer Res (2020) 15(26):3928–35. doi: 10.1158/1078-0432.CCR-20-0953 PMC8792803 32601075

[176] Ferrara N Adamis AP. Ten years of anti-vascular endothelial growth factor therapy. Nat Rev Drug Discovery (2016) 6(15):385–403. doi: 10.1038/nrd.2015.17 26775688

[177] Apte RS Chen DS Ferrara N. VEGF in signaling and disease: Beyond discovery and development. Cell (2019) 6(176):1248–64. doi: 10.1016/j.cell.2019.01.021 PMC6410740 30849371

[178] Zuazo-Gaztelu I Casanovas O. Unraveling the role of angiogenesis in cancer ecosystems. Front Oncol (2018) 8):248. doi: 10.3389/fonc.2018.00248 30013950 PMC6036108

[179] Thiel KW Devor EJ Filiaci VL Mutch D Moxley K Alvarez SA. TP53 sequencing and p53 immunohistochemistry predict outcomes when bevacizumab is added to frontline chemotherapy in endometrial cancer: An NRG Oncology/Gynecologic oncology group study. J Clin Oncol (2022) 28(40):3289–300. doi: 10.1200/JCO.21.02506 PMC9553389 35658479

[180] Aghajanian C Filiaci V Dizon DS Carlson JW Powell MA Secord AA. A phase II study of frontline paclitaxel/carboplatin/bevacizumab, paclitaxel/carboplatin/temsirolimus, or ixabepilone/carboplatin/bevacizumab in advanced/recurrent endometrial cancer. Gynecol. Oncol (2018) 2(150):274–81. doi: 10.1016/j.ygyno.2018.05.018 PMC6179372 29804638

[181] Makker V Colombo N Casado HA Santin AD Colomba E Miller DS. Lenvatinib plus pembrolizumab for advanced endometrial cancer. N Engl J Med (2022) 5(386):437–48. doi: 10.1056/NEJMoa2108330 PMC11651366 35045221

[182] Makker V Rasco D Vogelzang NJ Brose MS Cohn AL Mier J. Lenvatinib plus pembrolizumab in patients with advanced endometrial cancer: An interim analysis of a multicentre, open-label, single-arm, phase 2 trial. Lancet Oncol (2019) 5(20):711–8. doi: 10.1016/S1470-2045(19)30020-8 PMC11686814 30922731

[183] Hiraoka A Kumada T Kariyama K Takaguchi K Atsukawa M Itobayashi E. Clinical features of lenvatinib for unresectable hepatocellular carcinoma in real-world conditions: Multicenter analysis. Cancer Med (2019) 1(8):137–46. doi: 10.1002/cam4.1909 PMC6346240 30575325

[184] Hage C Hoves S Strauss L Bissinger S Prinz Y Pöschinger T. Sorafenib induces pyroptosis in macrophages and triggers natural killer Cell-Mediated cytotoxicity against hepatocellular carcinoma. Hepatology (2019) 4(70):1280–97. doi: 10.1002/hep.30666 31002440

[185] Li X Wang J Wang Q Luo T Song X Wan G. A novel VEGFR inhibitor ZLF-095 with potent antitumor activity and low toxicity. Heliyon (2023) 5(9):e15152. doi: 10.1016/j.heliyon.2023.e15152 PMC10209341 37251840

[186] Zhao Q Feng H Yang Z Liang J Jin Z Chen L. The central role of a two-way positive feedback pathway in molecular targeted therapies-mediated pyroptosis in anaplastic thyroid cancer. Clin Transl Med (2022) 2(12):e727. doi: 10.1002/ctm2.727 PMC8858618 35184413

[187] Marabelle A Le DT Ascierto PA Di Giacomo AM De Jesus-Acosta A Delord JP. Efficacy of pembrolizumab in patients with noncolorectal high microsatellite Instability/Mismatch Repair-Deficient cancer: Results from the phase II KEYNOTE-158 study. J Clin Oncol (2020) 1(38):1–10. doi: 10.1200/JCO.19.02105 PMC8184060 31682550

[188] Howitt BE Shukla SA Sholl LM Ritterhouse LL Watkins JC Rodig S. Association of polymerase e-Mutated and Microsatellite-Instable endometrial cancers with neoantigen load, number of Tumor-Infiltrating lymphocytes, and expression of PD-1 and PD-L1. JAMA Oncol (2015) 9(1):1319–23. doi: 10.1001/jamaoncol.2015.2151 26181000

[189] Talhouk A Derocher H Schmidt P Leung S Milne K Gilks CB. Molecular subtype not immune response drives outcomes in endometrial carcinoma. Clin Cancer Res (2019) 8(25):2537–48. doi: 10.1158/1078-0432.CCR-18-3241 30523022

[190] Wang Q Wang Y Ding J Wang C Zhou X Gao W. A bioorthogonal system reveals antitumour immune function of pyroptosis. Nature (2020) 7799(579):421–6. doi: 10.1038/s41586-020-2079-1 32188939

[191] Zhou W Liu H Yuan Z Zundell J Towers M Lin J. Targeting the mevalonate pathway suppresses ARID1A-inactivated cancers by promoting pyroptosis. Cancer Cell (2023) 4(41):740–56. doi: 10.1016/j.ccell.2023.03.002 PMC10085864 36963401

[192] Liu ZS Jing CL. A novel risk prediction model of pyroptosis-related genes for the prognosis and immunotherapy response of endometrial cancer. Eur Rev Med Pharmacol Sci (2022) 7(26):2259–78. doi: 10.26355/eurrev_202204_28456 35442481

[193] Huang X Li Y Li J Yang X Xiao J Xu F. The expression of Pyroptosis-Related gene may influence the occurrence, development, and prognosis of uterine corpus endometrial carcinoma. Front Oncol (2022) 12):885114. doi: 10.3389/fonc.2022.885114 35574367 PMC9103195

[194] Liu J Geng R Ni S Cai L Yang S Shao F. Pyroptosis-related lncRNAs are potential biomarkers for predicting prognoses and immune responses in patients with UCEC. Mol Ther Nucleic Acids (2022) 27):1036–55. doi: 10.1016/j.omtn.2022.01.018 PMC8844853 35228898

[195] Zhang C Bai J Yang Y Wang X Liu W Hou S. Construction of prediction model for prognosis of uterine corpus endometrial carcinoma based on pyroptosis gene. Clin Transl Oncol (2022) 25(5):1413–24. doi: 10.1007/s12094-022-03037-w 36520385

[196] Liu S Zeng C Lv H Zhang Y Xiong H Tang H. A novel defined Pyroptosis-Related gene signature for predicting the prognosis of endometrial cancer. Dis Markers (2022) 2022):7570494. doi: 10.1155/2022/7570494 36601599 PMC9806687

[197] Liang D Hu M Tang Q Huang M Tang L. Nine Pyroptosis-Related lncRNAs are identified as biomarkers for predicting the prognosis and immunotherapy of endometrial carcinoma. Int J Gen Med (2021) 14):8073–85. doi: 10.2147/IJGM.S338298 PMC8594792 34803394

